# Cost-effectiveness of community versus hospital eye service follow-up for patients with quiescent treated age-related macular degeneration alongside the ECHoES randomised trial

**DOI:** 10.1136/bmjopen-2016-011121

**Published:** 2016-10-24

**Authors:** M Violato, H Dakin, U Chakravarthy, B C Reeves, T Peto, R E Hogg, S P Harding, L J Scott, J Taylor, H Cappel-Porter, N Mills, D O'Reilly, C A Rogers, S Wordsworth

**Affiliations:** 1 Nuffield Department of Population Health, Health Economics Research Centre, University of Oxford, Oxford, UK; 2 Health Protection Research Unit in Gastrointestinal Infections, National Institute for Health Research, University of Oxford, Oxford, UK; 3 Centre for Experimental Medicine, Institute of Clinical Science, Queen's University Belfast, Belfast, UK; 4 Clinical Trials and Evaluation Unit, School of Clinical Sciences, University of Bristol, Bristol, UK; 5 NIHR BMRC at Moorfields Eye Hospital NHS Foundation Trust and UCL Institute of Ophthalmology, London, UK; 6 Department of Eye and Vision Science, Institute of Ageing and Chronic Disease, University of Liverpool, Liverpool, UK; 7 School of Social and Community Medicine, University of Bristol, Bristol, UK

**Keywords:** Macular degeneration, optometrists, ophthalmologists, cost-effectiveness

## Abstract

**Objectives:**

To assess the cost-effectiveness of optometrist-led follow-up monitoring reviews for patients with quiescent neovascular age-related macular degeneration (nAMD) in community settings (including high street opticians) compared with ophthalmologist-led reviews in hospitals.

**Design:**

A model-based cost-effectiveness analysis with a 4-week time horizon, based on a ‘virtual’ non-inferiority randomised trial designed to emulate a parallel group design.

**Setting:**

A virtual internet-based clinical assessment, conducted at community optometry practices, and hospital ophthalmology clinics.

**Participants:**

Ophthalmologists with experience in the age-related macular degeneration service; fully qualified optometrists not participating in nAMD shared care schemes.

**Interventions:**

The participating optometrists and ophthalmologists classified lesions from vignettes and were asked to judge whether any retreatment was required. Vignettes comprised clinical information, colour fundus photographs and optical coherence tomography images. Participants' classifications were validated against experts' classifications (reference standard). Resource use and cost information were attributed to these retreatment decisions.

**Main outcome measures:**

Correct classification of whether further treatment is needed, compared with a reference standard.

**Results:**

The mean cost per assessment, including the subsequent care pathway, was £411 for optometrists and £397 for ophthalmologists: a cost difference of £13 (95% CI −£18 to £45). Optometrists were non-inferior to ophthalmologists with respect to the overall percentage of lesions correctly assessed (difference −1.0%; 95% CI −4.5% to 2.5%).

**Conclusions:**

In the base case analysis, the slightly larger number of incorrect retreatment decisions by optometrists led to marginally and non-significantly higher costs. Sensitivity analyses that reflected different practices across eye hospitals indicate that shared care pathways between optometrists and ophthalmologists can be identified which may reduce demands on scant hospital resources, although in light of the uncertainty around differences in outcome and cost it remains unclear whether the differences between the 2 care pathways are significant in economic terms.

**Trial registration number:**

ISRCTN07479761; Pre-results.

**Strengths and limitations of this study**

- This is the first study to evaluate the cost-effectiveness of optometrist-led follow-up monitoring reviews for patients with quiescent treated neovascular age-related macular degeneration (nAMD) in community settings versus ophthalmologist-led monitoring reviews within the hospital eye service.
- Our results indicate that a cost-effective model of shared care between community optometrists and ophthalmologists in the management of nAMD may be achievable.
- Devolving responsibilities for monitoring patients to community optometrists may be a viable option to reduce the burden imposed on the hospital eye service by the new nAMD drugs.
- Given the ‘virtual’ nature of the trial, identification of the resource items and associated costs of providing follow-up monitoring reviews in community optometry practices was a particularly challenging task for optometrists. They had to refer to a hypothetical shared care scheme (ie, not currently routine), which may have affected the accuracy of some responses.
- The analysis used a 4-week time horizon, used mean imputation for missing data and made assumptions about the treatment pathway that would be followed if a shared care scheme was introduced in practice.

## Introduction

Neovascular age-related macular degeneration (nAMD) is a common disorder of the ageing eye, which if left untreated leads to severe central visual impairment. The current standard of care is treatment with biological drugs, which bind to or inhibit vascular endothelial growth factor (VEGF); these include ranibizumab (Lucentis) and bevacizumab (Avastin). These anti-VEGF therapies require intravitreal injections at 4–8-week intervals. Following multiple treatments, nAMD lesions become quiescent,^1^ ^2^ but there is a high risk of reactivation and regular review by experienced retinal specialists in hospital is routine clinical practice in many countries.^3–5^ This model of care results in ∼8–10 visits per year to a hospital eye service (HES) for most patients, which is expensive for healthcare systems and places a substantial burden on patients and their carers in terms of travel time and costs, and disruption to daily activities.^3–5^

Studies based in the UK estimate that total direct National Health Service (NHS) expenditure in eye health services totalled £2.15 billion in 2008,^6^ a figure which increased by 22.8% in 2013.^7^ Despite such an increase, the UK has the lowest ratio of consultant ophthalmologists per capita in the European Union,^8^ and this limited clinical capacity may undermine optimal care and timely access to potentially sight-saving treatments. For nAMD, much of the patient care discussion has centred on the expenditure allocated to the choice of anti-VEGF therapies,^2^ rather than the development of shared care options to address the increase in nAMD referrals to HES.

In glaucoma^9–11^ and diabetic retinopathy,^12^ ^13^ innovative shared care delivery models have been proposed and/or implemented, whereby non-medical eye care professionals, such as community optometrists (eg, high street opticians), provide some of the care, thereby alleviating the burden on the HES.^14^ ^15^ Devolving the monitoring of patients with quiescent nAMD disease to community optometrists may also be a clinically and economically viable solution to reduce burden on the HES, the patients and their carers. To the best of our knowledge, however, there is no evidence at present assessing such model of shared care. The ECHoES (Effectiveness of Community vs Hospital Eye Service) trial was, therefore, designed and undertaken to evaluate whether a shared care model between community optometrists and ophthalmologists for treatment of nAMD could be clinically effective and cost-effective. This paper reports the results of the cost-effectiveness analysis.

## Methods

The ECHoES trial was a ‘virtual’ non-inferiority randomised trial conceived to emulate a parallel group design. Optometrists and ophthalmologists made decisions about the reactivation status of lesions by reviewing vignettes, consisting of two sets of retinal images (baseline and index), including colour fundus and optical coherence tomogram (OCT) images, with accompanying clinical and demographic information, rather than by examining actual patients. In baseline images the lesion was quiescent; in index images the lesion could have been quiescent, suspicious or reactivated. Ninety-six participants (48 optometrists and 48 ophthalmologists) each assessed 42 vignettes in a randomised balanced incomplete block design; each of the 288 vignettes was assessed by 7 optometrists and 7 ophthalmologists (n=2016 total observations). Participants judged whether the lesion had reactivated or not in the index set of images. Full details of the trial methods are published elsewhere.^16^ ^17^

### Economic evaluation

A model-based cost-effectiveness analysis was conducted to compare the cost-effectiveness of optometrist-led follow-up monitoring reviews for patients with quiescent nAMD in community settings versus HES ophthalmologist-led monitoring appointments. The evaluation was undertaken using established guidelines for conducting and reporting cost-effectiveness analyses.^18^ ^19^ The perspective for the analysis was that of the UK NHS, following National Institute for Health and Care Excellence (NICE) recommendations.^18^ Owing to the virtual nature of the trial, it was not possible to collect data on patients'/carers' time off work/usual activities and travel costs to undergo eye assessment to inform a wider societal perspective. The economic evaluation assessed the cost per ‘correct’ retreatment decision, with ‘correct’ indicating that the reference standard lesion classification (as based on the judgements of three medical retina experts^16^) and the trial participants' judgements on the status of the lesion coincided. Since monitoring visits are typically conducted at 4-week intervals, the time horizon for our analysis was 4 weeks after the consultation in which patients were assessed by either an ophthalmologist or an optometrist to allow inclusion of downstream costs from retreatment decisions (eg, the costs of administering an anti-VEGF injection). Any costs and health consequences arising from incorrect treatment decisions beyond this 4-week time horizon were assumed to be captured within our measure of effectiveness (the number of correct retreatment decisions). No data are available on the effect of delaying anti-VEGF treatment after reactivation and modelling the costs and health consequences from incorrect retreatment decisions would therefore have relied entirely on assumptions or expert opinion. Owing to the short time horizon for analysis, discounting was not applied to costs or effects. We assumed that the costs and retreatment decisions would be constant over time and not vary with the number of previous monitoring consultations, as we are unaware of any reason why the incremental cost of monitoring or the probability of optometrists and ophthalmologists making correct retreatment decisions would be any different for the second or subsequent monitoring consultations than it would for the first consultation.

### Measurement and valuation of resource use

Measurement and valuation of resources used to perform a monitoring review in community optometric practices, required designing a bespoke questionnaire (available in online supplementary material—Resource use and cost questionnaire), which was completed by optometrists in the ECHoES trial. The questionnaire was developed by the ECHoES trial team (health economists, clinicians and optometrists) with external input from community optometrists not participating in the trial.

10.1136/bmjopen-2016-011121.supp1 Supplementary data

The resource use questionnaire asked respondents to describe the types and quantities of resources used for an ‘average’ monitoring review, which included staff time and equipment items, and report their unit costs (staff salaries and cost of equipment). As the trial was virtual, the optometrists also had to anticipate what resource items they did not currently have in the practice but which they would need to provide the monitoring service. In order to capture the cost of setting up facilities and purchasing/maintaining equipment required for the new service, participants were asked to provide details of any refurbishment work that would be required and for details of all necessary equipment, including its cost and annual service cost. The cost of equipment was annuitised using a 3.5% discount rate^20^ based on assumptions about the likely life span of equipment, then divided by the estimated number of potential patients that participating optometrists estimated would use the service annually. Costs associated with training trial participants included the 2-hour webinar^16^ ^17^ provided as part of the trial, time spent by participants reviewing the webinar and consulting other resources (as reported in the participants' questionnaire), and time spent by the ECHoES trial clinicians in preparing and delivering the webinar training (clinicians' self-report). Details are reported in online supplementary tables A.1 and A.2 in the online supplementary material. Value-added tax was excluded from costs as recommended.^18^ ^20^

The costs associated with ophthalmologists performing the monitoring assessments and the cost of administering intravitreal injections were based on data from the UK Inhibition of VEGF in Age-related choroidal Neovascularisation (IVAN) trial, in which a very detailed microcosting study was undertaken.^21^ ^22^ The consultation costs taken from IVAN were adjusted for inflation using the Hospital and Community Health Services Pay and Prices Index 2010/2011 and 2012/2013.^23^

### Estimation of total costs and cost-effectiveness across the treatment pathway

In order to generate an estimate of the total cost per correct retreatment decision, it was first necessary to develop an ‘acceptable’ model of shared-care between community optometrists and hospital ophthalmologists for co-management of follow-up appointments for patients with nAMD. The model was developed with the input of clinicians and optometrists in the ECHoES trial team, drawing from direct experiences of current clinical practice and participation in other similar models of shared care in eye health.

**Figure 1:**
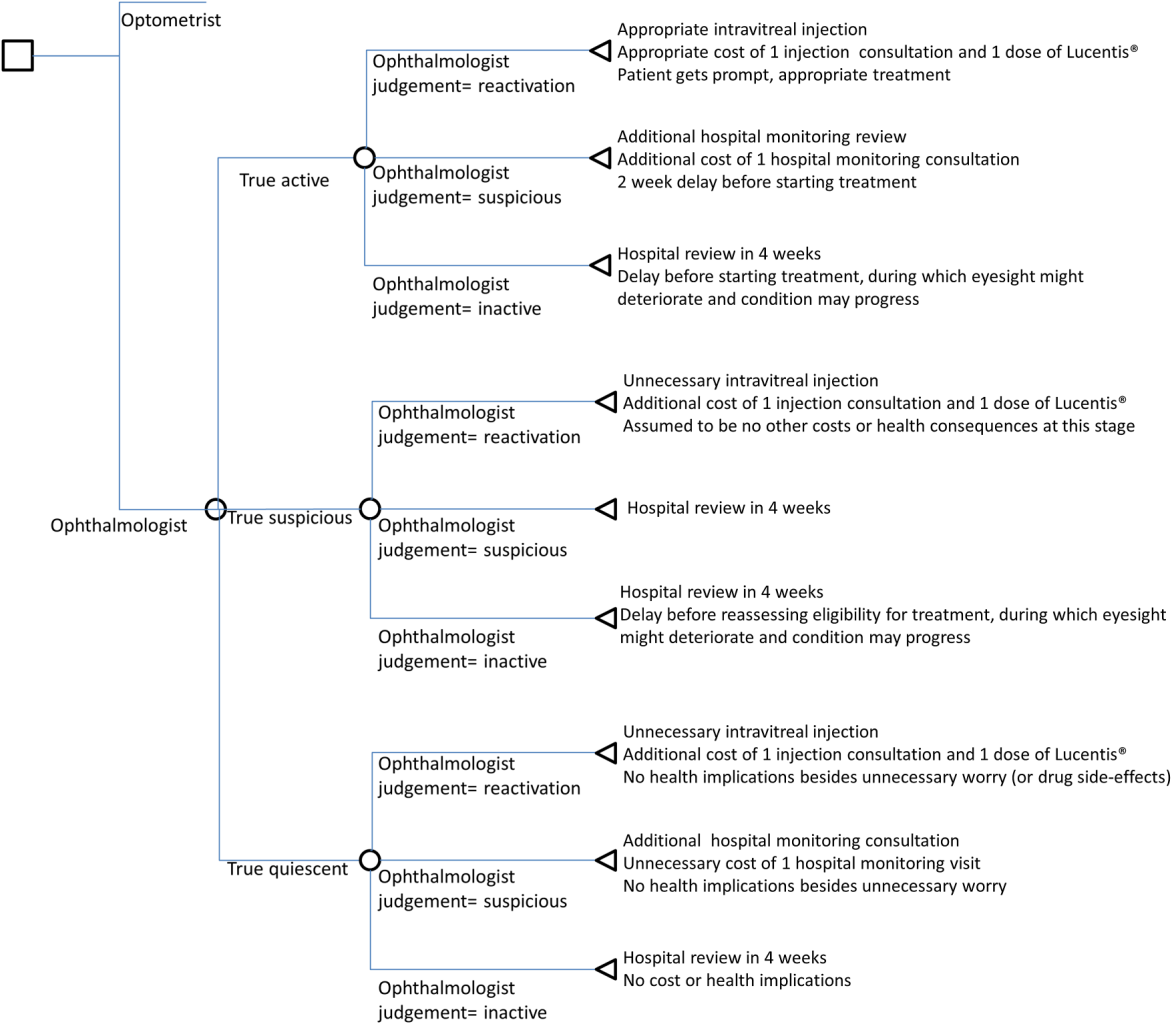
Decision tree for hospital ophthalmologist review.

**Figure 2:**
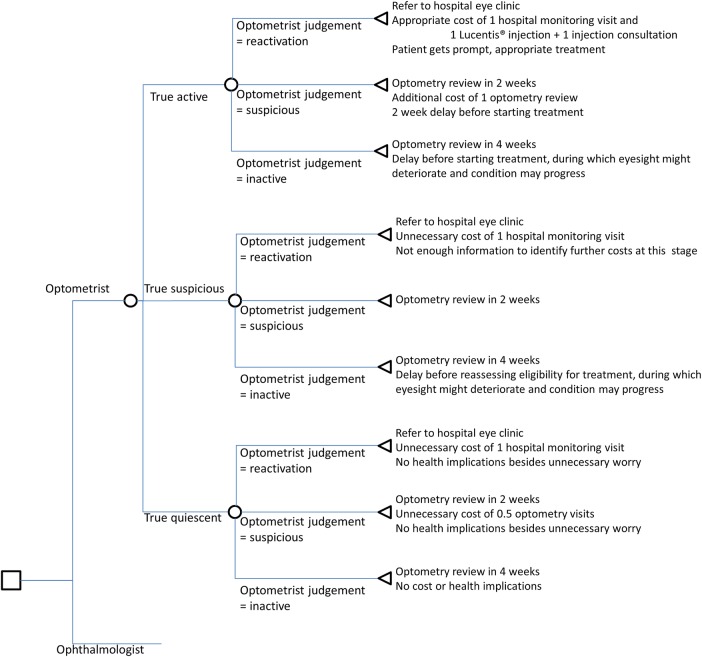
Decision tree for community optometrist review.

The care pathway followed by ophthalmologists in the HES reflected clinical practice in hospital settings at the time the trial was conducted (figure 1). The care pathway followed by community optometrists (figure 2) assumed that patients who were referred back to the HES with what community optometrists judged to be ‘reactivated lesions’, would attend a further HES monitoring review before the intravitreal injection. The rationale behind this choice was that, especially in the initial phases of implementation of the new shared care model, a second clinical opinion could help identify patients mistakenly classified as having reactivated lesions by cautious optometrists, avoiding unnecessary expensive treatments. For simplicity, we assumed that this additional consultation would identify all quiescent or suspicious lesions that had been incorrectly referred by optometrists. We acknowledge that we made certain assumptions and this is a limitation of our present study, however, modelling the sensitivity and specificity of this HES review would have complicated the analysis and required additional assumptions, which, in the absence of any empirical evidence, could be only speculative.

Having established the new hypothetical model of shared care, we developed the two decision trees (figures 1 and 2). These show pathways that would possibly be generated from the treatment assessments in the study, based on participants' classification of each vignette and the reference standard lesion classification. The reference standard lesion classification was the benchmark to establish whether the optometrists' and ophthalmologists' in the trial correctly judged the lesion status of each vignette. The model allowed for the short-term cost consequences of incorrect retreatment decisions by both optometrists and HES ophthalmologists. The lesion judgements made by the groups of ophthalmologists and optometrists in the study were then used to populate the model, and the associated costs for different pathways were calculated. In this way an average cost for each alternative care pathway was generated. An ‘incorrect’ decision implied that patients would have missed an opportunity for prompt treatment, had an unnecessary repeat monitoring appointment, or would have received unnecessary anti-VEGF injections.

Online supplementary tables A.1 and A.2 in the online supplementary material summarise the unit costs that were attached to the resource use information. The base case analysis assumed that ranibizumab (Lucentis) would be the drug of choice; its cost was £742 per dose.^24^ The ECHoES costing questionnaire was sent to 61 optometrists (who completed webinar training). So as to not overburden participants, the costing questionnaire was not a compulsory section, which reduced the number of returned questionnaires to 40 out of the 61 questionnaires (66%). Fifty-five of the 61 invited optometrists (90%) replied to a separate feedback questionnaire, which, in addition to some questions about how the training provided was received by participants, also contained information on the time spent on training by optometrists. To estimate the cost per monitoring review for each community optometry practice, information from the costing and the feedback questionnaires were merged using the participant's unique and anonymised identifier. Seven per cent of data from the completed questionnaires were missing (see online supplementary table A.3 in the online supplementary material). For consistency with the procedure adopted for costing consultations in the IVAN trial, mean values of the relevant variables were imputed whenever the information was missing. The total cost of a monitoring review performed in a community optometry practice, stratified by cost categories, is reported in online supplementary table A.4 in the online supplementary material. For the costs of the ophthalmologists' monitoring reviews in hospital, data from the IVAN trial were used, which provided 28 estimates of the cost of clinician-led HES monitoring reviews from different clinics at various hospitals. All costs are reported in 2013/2014 prices in British pounds (£).

In order to allow for the variability between clinics and imprecision around the mean cost per clinic, we followed an approach similar to that used in the IVAN trial^21^ to randomly assign consultation costs to each observation. Details of the random allocation procedure for costs are reported in the online supplementary material (‘Cost model—random allocation’ section). This approach propagates uncertainty around the costing into the economic evaluation, and ensures that the analysis is based on the average cost per consultation.

Given the large sample size and in the interest of simplicity, we used parametric methods based on the assumption of Gaussian distributions to estimate CIs and cost-effectiveness acceptability curves (CEACs).^25^ After calculating the costs for each vignette for each participant, we estimated regression models predicting costs and the probability of a correct retreatment decision as a function of whether the participant was an optometrist rather than an ophthalmologist, adjusting SEs for clustering by participant and correlations between costs and decision accuracy. The resulting SEs were used to calculate CIs, confidence ellipses and CEACs, which indicate the probability that the optometrist-led monitoring reviews are cost-effective compared with the ophthalmologist-led reviews for a range of potential threshold values that the NHS would be willing to pay for an additional unit of effect.^26^ Five sensitivity analyses tested different assumptions about the type of treatment received when a lesion was assessed as reactivated, and different shared care pathways between optometrists and ophthalmologists—given that the shared model of care is still hypothetical and may vary between hospitals (see the sensitivity analysis sections in the online supplementary material, supplementary tables A.5–A.14 and supplementary figures A.2–A.11 for further details):

1. Reactivated lesions were assumed to receive a course of three injections of ranibizumab at three injection consultations (vs one injection in the base case);
2. Reactivated lesions were assumed to receive one aflibercept injection during one injection consultation;
3. Reactivated lesions were assumed to receive one bevacizumab injection during one injection consultation;
4. Only the cost of a monitoring review was considered rather than the cost of the whole care pathway;
5. Assuming that an ophthalmologist would re-examine the OCT images taken by optometrists whenever the optometrist judgement was ‘lesion reactivated’ (rather a complete monitoring review at the HES as in the base case).

All analyses were undertaken using STATA V.12.1 (StataCorp, College Station, Texas, USA).

A post hoc χ^2^ test conducted in STATA was used to determine if there was a statistically significant difference in the proportion of truly quiescent vignettes that were classed as reactivated between ophthalmologists and optometrists.^16^ ^17^

## Results

### Resource use and costs

Online supplementary table A.4 in the online supplementary material reports the total cost of a monitoring review performed in a community optometry practice, stratified by cost categories. In terms of equipment resources required to perform a monitoring review, 97.5% of community practices participating in the trial owned a colour fundus camera, but <50% had a projector or a retroilluminated light box for displaying an Early Treatment of Diabetic Retinopathy Study (ETDRS) visual acuity chart. OCT equipment was owned by 45% (18/40) of the optometrists who completed the questionnaires. The average floor space of a community practice was reported as being 173 m^2^ (interquantile range=97) comprising, on average, 3.5 rooms (SD=2.20). Just over half of the respondents reported that their premises would require modifications to assess patients with nAMD, but that these would mainly consist of modifying existing rooms, rather than radical structural changes involving building work.

In terms of staffing, optometrists stated that they would undertake most of the required tasks within a monitoring review, that is, take patient history, carry out the clinical examination, visual assessment (including administration of 1% tropicamide drops) and the final assessment. However, one-third of respondents stated that they would share other activities, such as undertaking colour fundus photography and OCTs, with pre-registration optometrists and other support staff.

The total average cost of an optometrist-led community monitoring review was £52 per review (SD=£8). The corresponding average cost for an ophthalmologist-led HES monitoring review was £76 (SD=£44), as costed in the IVAN trial.^22^ These figures do not include the downstream costs after the monitoring review, which were part of the care pathway costs.

### Cost-effectiveness of monitoring by optometrists compared with ophthalmologists

Table 1 reports the care pathway cost, which depends on how ophthalmologists' and optometrists' judgements compare with the reference standard lesion classification. The pathway includes the cost of a monitoring consultation itself and also downstream costs whenever applicable (eg, ranibizumab injections and follow-up visits based on the care cost pathway decision tree).

**Table 1:** Care pathway costs—base case analysis

Lesion status assessment		Observations* (%)	Pathway cost† Mean (SD)
*Experts (true)*	*Optometrists' decision*		
Reactivated	Reactivated	795 (39.43)	£935.40 (45.50)
Reactivated	Suspicious	142 (7.04)	£103.61 (18.51)
Reactivated	Quiescent	57 (2.83)	£51.29 (9.08)
Suspicious	Reactivated	10 (0.50)	£118.12 (16.39)
Suspicious	Suspicious	11 (0.55)	£57.04 (9.10)
Suspicious	Quiescent	14 (0.69)	£52.96 (9.37)
Quiescent	Reactivated	105 (5.21)	£117.14 (32.61)
Quiescent	Suspicious	234 (11.61)	£78.31 (11.53)
Quiescent	Quiescent	648 (32.14)	£51.98 (8.23)
*Experts (true)*	*Ophthalmologists' decision*		
Reactivated	Reactivated	736 (36.51)	£ 882.67 (46.41)
Reactivated	Suspicious	196 (9.72)	£153.18 (92.25)
Reactivated	Quiescent	62 (3.08)	£77.01 (45.49)
Suspicious	Reactivated	1 (0.05)	£877.38 (N/A)
Suspicious	Suspicious	17 (0.84)	£68.84 (31.00)
Suspicious	Quiescent	17 (0.84)	£60.57 (17.16)
Quiescent	Reactivated	35 (1.73)	£882.29 (38.00)
Quiescent	Suspicious	146 (7.24)	£150.34 (95.19)
Quiescent	Quiescent	806 (39.98)	£75.28 (44.72)

*The number of observations (ie, vignettes) is 4038, namely 2016 retreatment decisions by optometrists and 2016 retreatment decisions by ophthalmologists.

†Pathway costs include the cost of a monitoring consultation and downstream costs (eg, injections and follow-up visits).

In terms of the 994 (795+142+57) vignettes rated as reactivated in the reference standard lesion classification, the optometrists made more correct decisions than the ophthalmologists (79.98% (795/994) compared with 74.04% (736/994)), and were less likely to misclassify reactivated lesions as suspicious or quiescent.^16^ The optometrists therefore showed greater sensitivity for identifying reactivated lesions.

Conversely, the optometrists had lower specificity and were three times more likely to incorrectly judge the 987 (105+234+648) truly quiescent vignettes as reactivated (10.63% (105/987)) than their clinical counterparts (3.55% (35/987), p<0.001 (χ^2^ test conducted in STATA)).

Table 2 reports the base case analysis of the cost-effectiveness of optometrists as compared with ophthalmologists in performing monitoring reviews, taking the average of the patient cost pathways described above. The mean care pathway cost for each assessment is similar between the two professional groups, equalling £411 for optometrists and £397 for ophthalmologists, and producing a cost difference of £13 (95% CI −£18 to £45). The non-significantly higher cost for optometrists is driven by the higher percentage of vignettes incorrectly classified as reactivated, thus incurring a larger cost for the healthcare service from an unnecessary ophthalmologist-led review. A slightly smaller percentage of correct decisions was made by optometrists (84.4%) compared with ophthalmologists (85.4%), a difference that was neither statistically significant (1.0%, 95% CI −4.5% to 2.5%) nor clinically meaningful.^16^

**Table 2:** Base case analysis of cost-effectiveness of a monitoring review performed by optometrists versus cost of a monitoring review performed by ophthalmologists

Costs and effects	Optometrists Mean (SD) (observations, n=2016)	Ophthalmologists Mean (SD) (observations, n=2016)
Cost of a monitoring review (pathway cost)	£410.78 (424.92)	£397.33 (387.46)
Percentage of correct assessments	84.4% (36.3%)	85.4% (35.3%)
Incremental cost (95% CI)	£13.45 (−£17.96 to £44.85)	
Incremental benefit, percentage of correct assessments (95% CI)	−1.0% (−4.5% to 2.5%)	
Incremental cost per correct assessment*	Optometrist-led care is dominated	

*The 95% CI around the incremental cost-effectiveness ratio could not be defined.

**Figure 3:**
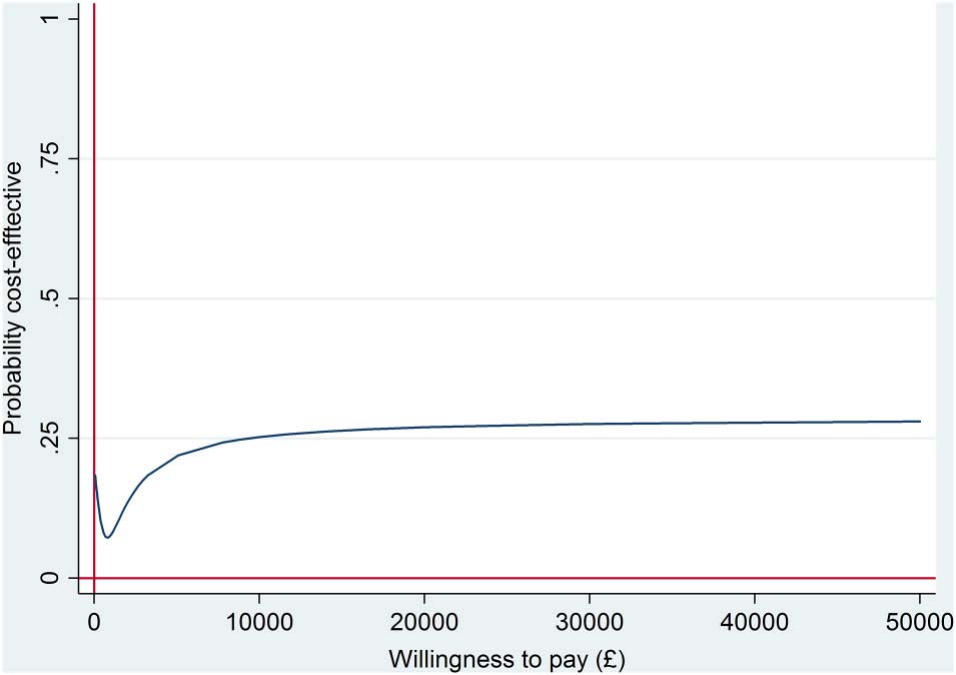
Cost-effectiveness acceptability curve for community optometry versus ophthalmologist-led care at a hospital eye service—base case analysis.

With non-significantly higher mean costs and non-significantly fewer correct treatment decisions, community optometry review is dominated by ophthalmologist-led reviews, being more costly and less effective. However, the differences are extremely small and not statistically significant: optometrist-led reviews increase the total costs by only £13 per review (3% of the total cost of the care pathway) and result in only one more incorrect decision per 100 monitoring reviews conducted. Furthermore, there remains uncertainty around this finding. This is illustrated by the cost-effectiveness plane in online supplementary figure A.1 (in the online supplementary material) and the CEAC in figure 3, which shows that the probability of optometrist-led reviews being cost-effective compared with ophthalmologist-led monitoring reviews is between 7% and 30% regardless of how much the NHS is willing to pay per correct retreatment decision.

Sensitivity analyses 1–3 confirm the results of the primary (base case) analysis (see online supplementary material), finding community optometry review dominated by ophthalmologist-led reviews. In contrast, sensitivity analysis 4 (which includes only the cost of the initial monitoring consultation and excludes all downstream costs of treatment and subsequent monitoring, see online supplementary material) indicates that if the NHS is willing to pay no more than £600 per correct retreatment decision, we can be 95% certain that optometrists-led reviews are a cost-effective option to ophthalmologist-led visits. It is important to emphasise, however, that sensitivity analysis 4 excludes the costs resulting from correct and incorrect decisions, which are an integral part of the costs associated with a retreatment decision. This highlights the importance of using even simple decision models for capturing all the relevant costs that pertain to a retreatment decision, so that final results are not biased. It also suggests that findings may be sensitive to the assumptions underlying the decision tree and, consequently, it becomes extremely important to develop reasonable models of shared-care delivery that are acceptable within current practice in community optometrist scenarios and hospital eye settings.

Optometrist-led care was also found to be less costly than monitoring by ophthalmologists in sensitivity analysis 5, in which patients referred by community optometrists were assumed to have a review of the pre-existing images, rather than a full hospital monitoring consultation. In this scenario, the care pathway for optometrist-led monitoring was found to cost £9 (95% CI -£39 to £22) less than ophthalmologist-led monitoring. Referring patients to receive monitoring at community optometry practices is therefore cost-effective in this scenario if the NHS is willing to accept one additional incorrect retreatment decision in order to save £870, and had a 69% probability of being cost-effective if the NHS were willing to pay £200 per correct retreatment decision.

## Discussion

This study suggests that community optometry-led follow-up reviews for patients with quiescent nAMD lesion is associated with non-significantly higher costs and marginally fewer correct treatment decisions compared with ophthalmologist-led care, although the difference in outcomes is not clinically meaningful^16^ and, when uncertainty around the joint distribution of costs and effects is taken into account, it remains unclear whether the differences between the two care pathways are economically significant.

The optometrists showed greater sensitivity for identifying reactivated lesions compared with ophthalmologists. Given the hypothesised model of shared care, the implication of this result is twofold. When an optometrist correctly judges the lesion as reactivated, the confirmatory ophthalmologist-led monitoring review assumed in the model will represent an unnecessary cost. In contrast, when an ophthalmologist judges a lesion to have reactivated, the model assumes that the patient will receive ranibizumab without the need of a second opinion: this avoids a further unnecessary monitoring review if the ophthalmologist's judgement is correct. When either optometrists or ophthalmologists mistakenly judge reactivated lesions to be quiescent, this will generate delays in treatment that may result in deterioration in patient eyesight. This clinically important outcome, which occurred more commonly for ophthalmologists, was not quantified in our analysis as it lay beyond the 4-week time horizon.

Conversely, the optometrists had lower specificity and were three times more likely to incorrectly judge truly quiescent vignettes as reactivated than their clinical counterparts. For optometrists, such mistakes result in an unnecessary ophthalmologists-led monitoring review. We assumed that this consultation would identify all incorrect referrals, thereby avoiding unnecessary treatment, although in practice some such referrals may be missed. In contrast, if an ophthalmologist makes the same error, the patient will undergo a costly treatment which is unnecessary at that moment in time, simply because there is no second check of the ophthalmologists' diagnosis.

Results were sensitive to the assumptions underpinning the proposed model of shared care. A ‘post hoc’ sensitivity analysis used a different care pathway, whereby patients referred by optometrists had a review, by ophthalmologists, of pre-existing OCT and colour images to assess eligibility for treatment, as a form of quality assurance, rather than a full hospital monitoring consultation. This analysis found that optometrist-led care could be cost-effective if the value that the NHS places on each correct retreatment decisions is <£870. Other care pathways could also be used, which are not modelled here.

Based on the prevalence and incidence of nAMD,^27–29^ around 219 000 patients currently attend VEGF clinics in England each month, of whom 52 000 (19%) have bilateral disease. We assumed that patients would be referred from the HES to community optometrists for monitoring if they did not meet the IVAN retreatment criteria in either eye 1 month after finishing a course of anti-VEGF treatment. This suggests that ∼21 950 patients in England may be eligible for referral to community optometry each month, equating to 535 550 community monitoring reviews per year, if patients are, on average, quiescent for 2 months. The costing analysis suggests that on average the initial monitoring consultation is £24 cheaper if performed by community optometrists rather than the HES, equating to initial savings of £12.7 million (£24×535 550) across NHS England. However, this figure takes no account of the costs after the patient is referred back to the HES for review and treatment. Allowing for the costs accrued across the entire treatment pathway using the base case analysis, community optometry is on average £13 more costly and referring patients for monitoring by community optometrists is expected to cost an additional £7.2 million (95% CI −£9.6 to £24.0 million) across England (£13 (95% CI −£18 to £45)×535 550). In contrast, using the alternative care pathway described in sensitivity analysis 5 (with review of images rather than a repeat monitoring consultation), community optometry could save £4.6 million (£9×535 550). However, there is substantial uncertainty around the incremental cost of shared care: within the base case analysis, there is a 20% chance that optometrist-led care could be cost-saving, compared with a 71% chance in scenario 5.

These findings have to be interpreted in light of some limitations of the study, resulting from the ‘virtual’ nature of the trial. First, costs were estimated using a hypothetical model of shared care developed with the input of clinicians and optometrists in the ECHoES trial team. Despite attempts to conceive the model in a way that could be as generalisable as possible, the shared care model assumed here may not be considered an adequate representation of all local circumstances/settings and therefore specific adaptations may be appropriate. Sensitivity analyses suggested that results were sensitive to some changes in the assumptions underpinning the model.

Second, the shared model is based on evaluating only one eye (a limitation of the trial; the IVAN trial did not collect frequent images for the fellow eyes, so vignettes based on information about both eyes could not be constructed). Considering bilateral disease would not change the sensitivity and specificity of decisions for each eye, but the rates of false-positive and false-negative *referrals* (at the level of a patient) would almost certainly be affected. More empirical data are needed on practitioners' referral decisions for patients with bilateral disease.

Third, there are limited data on whether the proportions of reactivated (49%), inactive (49%) and suspicious (2%) reference classifications among the vignettes are representative; varying the proportions does not change the sensitivity and specificity but does change the positive and negative predictive values, that is, the proportions of false positives and false negatives that are observed^16^ and may affect cost-effectiveness. Unfortunately, there is no direct information on these proportions in the course of usual care, and this may depend on the ‘treatment-free interval’ that is chosen before ‘discharging’ a patient with inactive disease to optometric review. Data from the UK nAMD Database^5^ are most relevant; the median time to retreatment after a 3-month treatment-free interval was 2.5 months (equivalent to reactivation at 40%). The treatment frequencies observed in the HARBOR and AURA studies^3^ ^4^ suggested that treatment was needed on more than 50% of the monthly visits.

Fourth, optometrists completing costing questionnaires had to answer questions on the resources that they would need to implement a hypothetical shared care scheme in their practice that is not currently routine, which may have affected the accuracy of some responses, especially with respect to volume of patients with nAMD that the practice may accommodate. Finally, in order not to overburden participants, the health economics questionnaire was not a compulsory section for participants, which reduced the number of completed resource use questionnaires. Mean imputation was also required due to item non-response. However, those who did complete the questionnaires varied substantially in terms of their practice size and type, capturing heterogeneity of optometry practice settings. The analysis also comprises a cost-effectiveness analysis with a short time horizon and an intermediate end point (correct retreatment decisions). This end point does not distinguish between false positives (ie, unnecessary referrals) and false negatives (ie, missed opportunities for timely treatment), which will have very different impacts on costs and health and may have different ceiling ratios. This could have affected the results, particularly as optometrists tended to have more false positives and fewer false negatives than ophthalmologists. Owing to the virtual trial setting, the analysis could not take into consideration benefits and costs accrued to patients and their carers.

Part of the trial inclusion criteria for optometrists was that they should not have had any prior exposure to retina clinics or in-depth knowledge of macular morphology. This was to ensure that we were evaluating a group of optometrists that were similar to most community optometrists. While we did provide some training in the trial, this training was short. When considering the result of non-inferiority, it is notable that the ophthalmologist participants in the trial represented usual care in the NHS. Both sets of participants were compared with the reference standard who were retina specialists recognised in their field as experts in this condition. Therefore, we can only speculate about what might happen to error rates, and the difference in the types of error made, with increasing experience in the groups studied. Without feeding back expert classifications, we see no reason for any difference in the error rates or types of error. With feedback, one might expect the performance of both groups to improve, but we think that the difference in the rates of different types of error might be maintained, given the different settings (primary vs secondary care) in which decisions would be made.

A further point, which might support the introduction of a model of shared care, is the consideration of patient views for altering the balance of care. The travel costs and time away from usual activities would most likely be less with visits to a community optometrist and this option might be preferred by patients. Although patient's preferences and costs were not directly included in our analyses, qualitative work undertaken as part of the ECHoES trial reported that there was enthusiasm among health professionals and service users about the possibility of shared care for nAMD, as it was felt to have the potential to relieve HES burden and represent a more patient-centred service.^30^

In summary, the base case analysis found that correct retreatment decisions by optometrists were slightly less common compared with ophthalmologists, leading to marginally higher costs for the former—although not statistically significant. Sensitivity analyses that reflected different practices across eye hospitals, indicated that shared care pathways can be identified which may represent cost-effective models of shared care between community optometrists and ophthalmologists in the management of nAMD, although in light of the uncertainty around differences in outcome and cost it remains unclear whether the differences between the two care pathways are significant in economic terms. The present study has allowed the various potential ‘ingredients’ of a future shared care model to be costed and represents an important contribution to innovatively shape clinical practice. ECHoES demonstrates that shared care will be non-inferior to current care in the HES and with repeated exposure to clinical situations and appropriate feedback community optometrists' skills can only improve. Harnessing this body of skilled community-based workforce has the potential to address the serious shortages of specialist staff in the HES. This is particularly timely because the UK NHS not only has the lowest rate of ophthalmologists per capita within Europe^8^ but also treats more patients with nAMD more intensively than in other countries.^31^ This, on the one hand, allows some of the best functional outcomes in Europe^4^ but, on the other hand, makes an already burdensome workload even less manageable. Thus, expanding the clinical role of non-medical staff, after appropriate training, is a promising strategy to meet the challenge of shortage of ophthalmologists in the HES. Further research could assess the applicability of the proposed model of shared care to other retinal diseases such as diabetic macular oedema and retinal vein occlusion. In particular, data are required on the downstream clinical pathways after optometrist assessment and likely patient numbers who could participate in such models.
