# Supplementary material for: Cost-effectiveness of community versus hospital eye service follow-up for patients with quiescent treated age-related macular degeneration alongside the ECHoES randomised trial
Source: BMJ Open. 2016 Oct 24;6(10):e011121. doi: 10.1136/bmjopen-2016-011121 (PMC5093395; doi:10.1136/bmjopen-2016-011121)
Supplement: Supplementary data [file bmjopen-2016-011121supp.pdf]

SUPPLEMENTARY MATERIAL

The cost-effectiveness of community versus hospital eye service follow-up for patients with quiescent treated age-related macular degeneration alongside the ECHoES randomised trial

Violato M, Dakin H, Chakravarthy U, Reeves BC, Peto T, Hogg RE, Harding SP, Scott LJ, Taylor J, Cappel-Porter H, Mills N, O'Reilly D, Rogers CA, Wordsworth S

Supplementary Table A.1 – ECHoES trial unit costs (all costs in 2013/2014)

Item	Unit Cost (£)	Source	Notes
ETDRS visual acuity charts - 4 metres viewing distance required	£83	Expert's opinion[a], recommended website: http://sussexvision.co.uk/index.php/distance-tests/logmar-charts/4-metre-viewing/logmar-4m-etdrs-chart-r-original.html (accessed 30/09/14)	Sussex vision item. ECHoES protocol requires three of them: one for doing the refraction, one for the right eye and one for the left eye.
Projector that includes ETDRS chart	£2,850	Personal communication (email) with Topcon Country Sales Manager Ireland, Topcon Europe BV T/A Topcon Ireland, Dublin: model 1240263 CC-100XP LED with remote control (29/09/14)	Model 1240263 CC-100XP LED with remote control
Retro-illuminated light box	£878	Expert's opinion[a], recommended website: http://sussexvision.co.uk/index.php/distance-tests/logmar-test-types/precision-vision-logmar-cabinet.html (accessed 30/09/14)	Sussex vision item
Bulb for retro-illuminated light box	£15	Expert's opinion: http://sussexvision.co.uk/index.php/distance-tests/logmar-test-types/tube-for-sdt-396-lpv-cabinet-without-diffuser.html (accessed 30/09/14)	Sussex vision item. Two bulbs, which needs to be replaced every two years, for each chart.
Trial frame	£375	Expert's opinion[a], recommended website: http://www.opticalmarketplace.co.uk/new-equipment/optical-	Optical market place

		equipment/trial-frames/omp1559/oculus-ub4-trial-frame/ (accessed 30/09/14)	
Lens set	£475	Expert's opinion[a], recommended website: http://www.opticalmarketplace.co.uk/new-equipment/optical-equipment/trial-lens-sets/omp1464/quality-trial-lens-set/ (accessed 30/09/14)	Optical market place item
Light meter to measure luminance. e.g. SPER Scientific	£120	Expert's opinion[a], recommended website: http://www.coleparmer.co.uk/Product/Sper_Scientific_840006_Light_Meter_with_Analog_Output/UY-01588-24?referred_id=3482&gclid=CKX6m8PjiMECFUn3wgodxJYA6Q (accessed 30/09/14)	Cole Parmer item
Slit-lamp	£4000	Expert's opinion[a]	Personal communication (email) with Dr Ruth Hogg, Institute of Clinical Science, Queen's University Belfast, 2014 (30/09/2014)
Computer	£549	Dell Corporation Limited is registered in England and Wales. Company Registration Number: 2081369. Registered address: Dell House, The Boulevard, Cain Road, Bracknell, Berkshire, RG12 1LF, UK. http://www.dell.com/uk/business/p/desktops-n-workstations.aspx?c=uk&l=en&s=bsd&~ck=mn (accessed 01/10/14)	Dell desktop OptiPlex 9020
Computer network	£1000	Expert's opinion confirming estimates at	Guide price to build a simple network of up to ten computers

		http://www.itdonut.co.uk/it/communications/networking (accessed 29/09/14)	
Printer	£155	Dell Corporation Limited is registered in England and Wales. Company Registration Number: 2081369. Registered address: Dell House, The Boulevard, Cain Road, Bracknell, Berkshire, RG12 1LF, UK. http://accessories.euro.dell.com/sna/sna.aspx?c=uk&cs=ukdhs1&l=en&s=dhs&~topic=printer_shopall_lasers (accessed 1/10/14)	Dell start price for C1660w Colour Printer
Eye drops, tropicamide 1%	£0.50	British National Formulary (BNF) [1] https://www.medicinescomplete.com/mc/bnf/current/PHP6943-minims-tropicamide.htm?q=minims%20tropicamide&t=search&ss=text&p=1#PHP6943-minims-tropicamide (accessed 06/11/14)	Single use - Net price 20 × 0.5 mL = £10.00
Consultant	£139	Personal Social services Research Unit. Units Costs of Health and Social Care 2013. University of Kent.	Table 15.5 Consultant: medical. Cost including Qualifications.
Ranibizumab (Lucentis®)	£742.17	British National Formulary (BNF)[1] https://www.medicinescomplete.com/mc/bnf/current/PHP7170-lucentis.htm?q=ranibizumab&t=search&ss=text&p=2#PHP7170-lucentis (accessed 12/11/2014)	Ranibizumab (Lucentis®)
Eylea	£816	British National Formulary (BNF)[1] https://www.medicinescomplete.com/mc/bn	Eylea

		f/current/PHP19020-eylea.htm?q=Eylea&t=search&ss=text&p=1#PHP19020-eylea (accessed 14/11/14)	
Bevacizumab (Avastin®)	£49	Dakin HA, Wordsworth S, Rogers CA, Abangma G, Raftery J, Harding SP, Lotery AJ, Downes SM, Chakravarthy U, Reeves BC; IVAN Study Investigators. Cost-effectiveness of ranibizumab and bevacizumab for age-related macular degeneration: 2-year findings from the IVAN randomised trial. *BMJ Open*. 2014 Jul 29;4(7):e005094. doi: 10.1136/bmjopen-2014-005094.	Bevacizumab (Avastin®)
**Ratio**			
**Type of ratio**	**Ratio**	**Source**	**Notes**
ratio 'salary oncost/salary'	0.234	Personal Social services Research Unit. Units Costs of Health and Social Care 2013. University of Kent[2].	Table 9.1 Community physiotherapist. Ratio applied to salary as reported by participant to the Health Economics Questionnaire
ratio 'qualification/salary'	0.237		
ratio 'overheads/salary'	0.756		
ratio 'capital overheads/salary'	0.093		

a Expert's opinion from personal communication (Dr Ruth Hogg, Institute of Clinical Science, Queen's University Belfast, 2014).

Supplementary Table A.2 – Salary of staff employed in optometrist practice

Salary Band	Optometrist	Pre-registration optometrist	Optical assistant	Clerical/retailer staff	Practice manager	Other administrative staff
Less than £20,000	4	5	24	14	3	10
£20,000 to £29,999	2	0	4	2	5	2
£30,000 to £39,999	9	0	0	0	6	0
£40,000 to £49,999	9	0	0	0	0	0
£50,000 to £59,999	10	0	0	0	0	0
£60,000 to £69,999	4	0	0	0	0	0
£70,000 to £79,999	1	0	0	0	0	0
£80,000 per year or more	1	0	0	0	0	0
Observations	*40*	*5*	*28*	*16*	*14*	*12*

Supplementary Table A.3 – Missing answers on key resource items in the optometrists' questionnaire

Resource item	Percentage of missing answers (n/N)
**Equipment for monitoring review[a]**	
ETDRS visual acuity charts	0 (0/40)
Projector that includes ETDRS chart	2.5% (1/40)
Retro-illuminated light box	2.5% (1/40)
Trial frame	0 (0/40)
Lens set	0 (0/40)
Light meter to measure luminance	0 (0/40)
Slit-lamp	0 (0/40)
Colour fundus camera	2.5% (1/40)
OCT acquisition system	7.5% (3/40)
OCT acquisition system with fundus photography included as a component	5% (2/40)
Computer	2.5% (1/40)
Computer network	0 (0/40)
Printer	0 (0/40)
**Duration of tasks in monitoring review[a]**	
Taking patient history	0 (0/40)
Clinical examination: Slit lamp biomicroscopy, anterior segment and macula	0 (0/40)
Visual acuity assessment	5% (2/40)
Administration of 1% tropicamide drops	7.5% (3/40)
Colour fundus photography (or equivalent colour fundus image)	32.5% (13/40)
Spectral domain optical coherence tomography (OCT)	32.5% (13/40)
Final assessment	0 (0/40)
Update patient record	22.5% (9/40)
**Time spent by optometrists revisiting webinars[b]**	4% (2/55)
**Time spent by optometrists consulting other resources[b]**	31% (17/55)

[a] So as to not overburden participants the health economics questionnaire was not a compulsory section for participants, which reduced the number of returned questionnaires to 40 out of the 61 questionnaires initially sent out to optometrists.
[b] So as to not overburden participants the feed-back questionnaire was not a compulsory section for participants, which reduced the number of returned questionnaires to 55 out of the 61 questionnaires initially sent out to optometrists.

Seven percent of data from the completed questionnaires were missing, although the majority of missing data were associated with tasks for which many optometrists may have limited experience (e.g. conducting colour fundus photography, OCT or updating medical records). Given the structure of the dataset, it would have been extremely difficult to conduct multiple imputation and this would have added additional complexity into the assignment of costs. We therefore used mean imputation to allow for missing data, following the approach used to cost up consultations in the IVAN trial [3].

Supplementary Table A.4 - Cost of a monitoring review performed by community optometrists (authors' calculations)

Component of cost per monitoring review	Mean cost, £(SD)
Equipment	£22.99 (5.55)
Refurbishment/building/rent	£0.05 (0.15)
Staff labour	£27.26 (7.32)
Preparation and delivery of webinar training	£0.13 (0.10)
Optometrist's time on training	£0.89[#] (1.08)
Eye drops, tropicamide 1%	£0.50 (0.00)
**Total cost per monitoring review**	**£51.82 (8.15)**

[#] The cost of training was calculated as the cost of attending (£124 (SD 69.26)), revisiting (£96 (SD 111.58) webinars, and consulting other resources (£66 (SD 112.35)), as reported by the optometrists in the questionnaires, divided by the average annual number of patients (321 (SD 188)) that a practice would be able to accommodate, as reported by each optometrist in the questionnaire. This gives the figure of 89p per consultation ((124+96+66)÷321).

COST MODEL –RANDOM ALLOCATION

Following the methods adopted in the IVAN trial, we randomly sampled from the distribution of costs from different optometrist practices using the following procedure:

- A weight was assigned to each practice based on the number of patients they could accommodate monthly, after any necessary changes to their practice are implemented, divided by the total number of all patients with quiescent nAMD that could be accommodated by all the practices in the study. Cumulative weights were then calculated and assigned to each optometric practice.
- For each vignette (potential patient) a random number was generated. This random number determined which of the monitoring review costs each vignette (i.e. each potential patients) was randomly assigned to according to the following decision rule: if the random number assigned to the first vignette (potential patient) was X and the weight assigned to the first optometry practice was A and the weight assigned to the second optometry practice was B, the vignette was assigned the cost from the first optometry practice if X<A and was assigned the cost for the second practice if A<X<A+B.
- All the monitoring consultations associated to that vignette (potential patient) were valued at the price for that monitoring review.

In this way, for each vignette (potential patient) addressed by a community optometrist, we randomly drew a value for the cost of the community optometry review from the distribution of ECHOES monitoring review costs; for each vignette addressed by an ophthalmologist, we randomly drew a value for the cost of a hospital review from the cost distribution used in IVAN. For those vignettes where a wrong decision results in an additional hospital monitoring consultation, or an injection, we drew additional random numbers to sample those consultation costs from the distribution of costs as reported in IVAN.

ADDITIONAL FIGURE ON THE BASE CASE ANALYSIS

Supplementary Figure A.1 – Optometrists vs opththalmogists cost-effectiveness plane – base case scenario, showing the outer 95% confidence ellipse in blue and the 85% confidence ellipse (the widest confidence interval around the ICER that is defined) in brown. The green line shows the tangent to the 85% confidence ellipse.

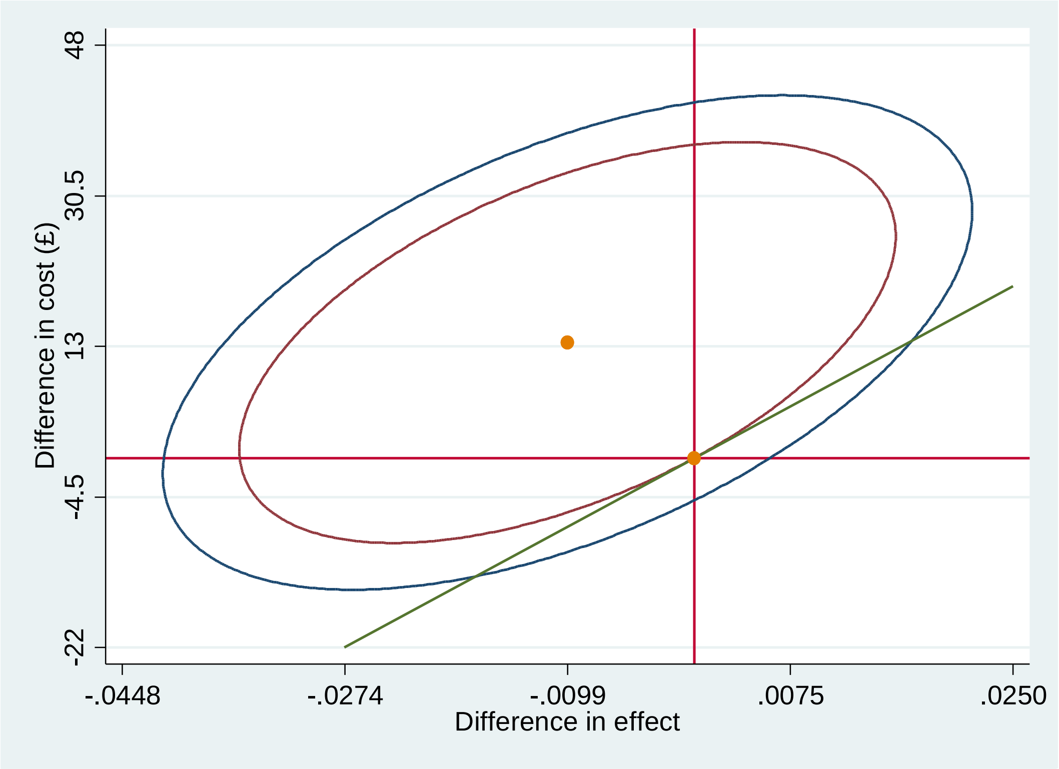

SENSITIVITY ANALYSES

Sensitivity analyses were conducted to explore the effect of varying the way of delivering treatment for lesions assessed as reactivated, which is one of the main cost drivers in our analysis. In the base case analysis, it was assumed that treatment for an active lesion consisted of one ranibizumab injection given during an injection consultation.

Sensitivity Analyses 1, 2, and 3

In our first three sensitivity analyses, one ranibizumab injection was replaced with alternative treatments to reflect different practice across eye hospitals (sensitivity analyses 1 and 2) and to make a comparison with a much cheaper drug assessed in the IVAN trial (sensitivity analysis 3). More specifically:

- Sensitivity analysis 1: treatment for an active lesion was assumed to be a course of three injections of ranibizumab at three subsequent injection consultations, with no additional monitoring reviews;
- Sensitivity analysis 2: treatment for an active lesion was assumed to be given in the form of one aflibercept injection during an injection consultation;
- Sensitivity analysis 3: treatment for an active lesion consisted of one bevacizumab injection given during an injection consultation.

Care pathway costs (**Supplementary Tables A.5**, **A.7**, and **A.9**), cost-effectiveness of a monitoring review performed by optometrists versus a monitoring review performed by ophthalmologists

(**Supplementary Tables A.6**, **A.8**, and **A.10**), **cost-effectiveness planes (Supplementary Figures A.2, A.4, A.6)**, and cost-effectiveness acceptability curves (**Supplementary Figures A.3**, **A.5**, and **A.7**), are reported below for each of the three sensitivity analyses. Similar to the base case analysis, optometrist-led monitoring reviews remained dominated by ophthalmologist-led reviews, and there was no acceptable willingness-to-pay threshold for which we can be 95% confident that the two alternative ways of performing a monitoring review differ in value.

Results of sensitivity analysis 1: Course of 3 ranibizumab injections

Supplementary Table A.5: Care pathway costs – sensitivity analysis 1

**Lesion status assessment**		**Obs[1] (%)**	**Pathway cost[2] (£) Mean (SD)**
**Experts (true)**	**Optometrists' decision**		
Reactivated	Reactivated	795 (39.43)	£2548.83 (67.90)
Reactivated	Suspicious	142 (7.04)	£103.61 (18.51)
Reactivated	Quiescent	57 (2.83)	£51.29 (9.08)
Suspicious	Reactivated	10 (0.50)	£118.12 (16.39)
Suspicious	Suspicious	11 (0.55)	£57.04 (9.10)
Suspicious	Quiescent	14 (0.69)	£52.96 (9.37)
Quiescent	Reactivated	105 (5.21)	£117.14 (32.61)
Quiescent	Suspicious	234 (11.61)	£78.31 (11.53)
Quiescent	Quiescent	648 (32.14)	£51.98 (8.23)
**Experts (true)**	**Ophthalmologists' decision**		
Reactivated	Reactivated	736 (36.51)	£2495.81 (70.01)
Reactivated	Suspicious	196 (9.72)	£153.18 (92.25)
Reactivated	Quiescent	62 (3.08)	£77.01 (45.49)
Suspicious	Reactivated	1 (0.05)	£2452.74 (N/A)
Suspicious	Suspicious	17 (0.84)	£68.84 (31.004)
Suspicious	Quiescent	17 (0.84)	£60.57 (17.16)
Quiescent	Reactivated	35 (1.73)	£2493.45 (65.87)
Quiescent	Suspicious	146 (7.24)	£150.34 (95.19)
Quiescent	Quiescent	806 (39.98)	£75.28 (44.72)

[1]The number of observations (i.e. vignettes) is 4032, namely 2016 referring to Optometrists and 2016 referring to Ophthalmologists.
[2]Pathway cost includes the cost of a monitoring consultation and downstream costs (e.g. injections and follow-up visits)

Supplementary Table A.6: Cost-effectiveness – Cost-effectiveness of a monitoring review performed by optometrists versus a monitoring review performed by ophthalmologists – sensitivity analysis 1

**Costs and effects**	**Optometrists Mean (SD) (n=2016)**	**Ophthalmologists Mean (SD) (n=2016)**
Cost of a monitoring review (pathway cost)	£1,047.03 (1,213.05)	£1,015.01 (1,168.80)
Percentage of correct assessments	84.4% (36.3%)	85.4% (35.3%)
Incremental cost (95% CI)	£32.02 (-£60.87, £124.90)	

Incremental benefit, percentage of correct assessments (95% CI)	-1.0% (-4.5%, 2.5)
Incremental cost per correct assessment[1]	Optometrist-led care is dominated

[1]The 95% CI around the ICER could not be defined

Supplementary Figure A.2 – Optometrists vs opththalmogists cost-effectiveness plane for sensitivity analysis 1, showing the outer 95% confidence ellipse in blue and the 81% confidence ellipse (the widest confidence interval around the ICER that is defined) in brown. The green line shows the tangent to the 81% confidence ellipse.

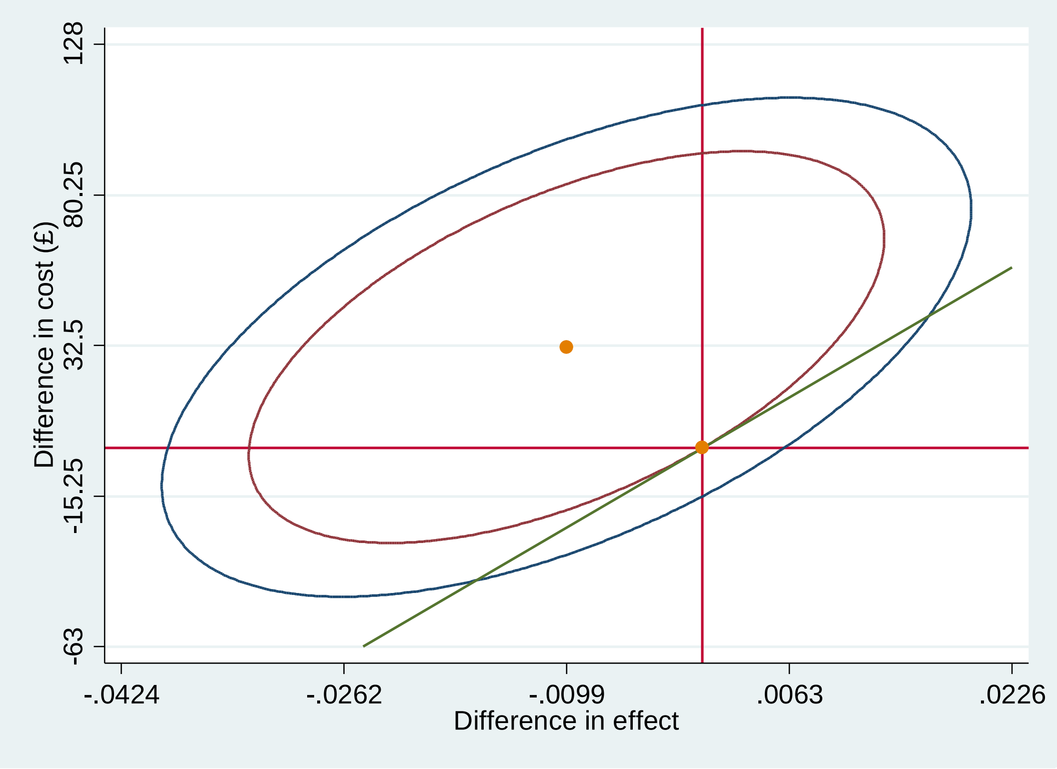

Supplementary FigureA.3: Cost-effectiveness acceptability curve showing the probability that the intervention is cost-effective at different willingness-to-pay thresholds – Optometrists versus Ophthalmologists

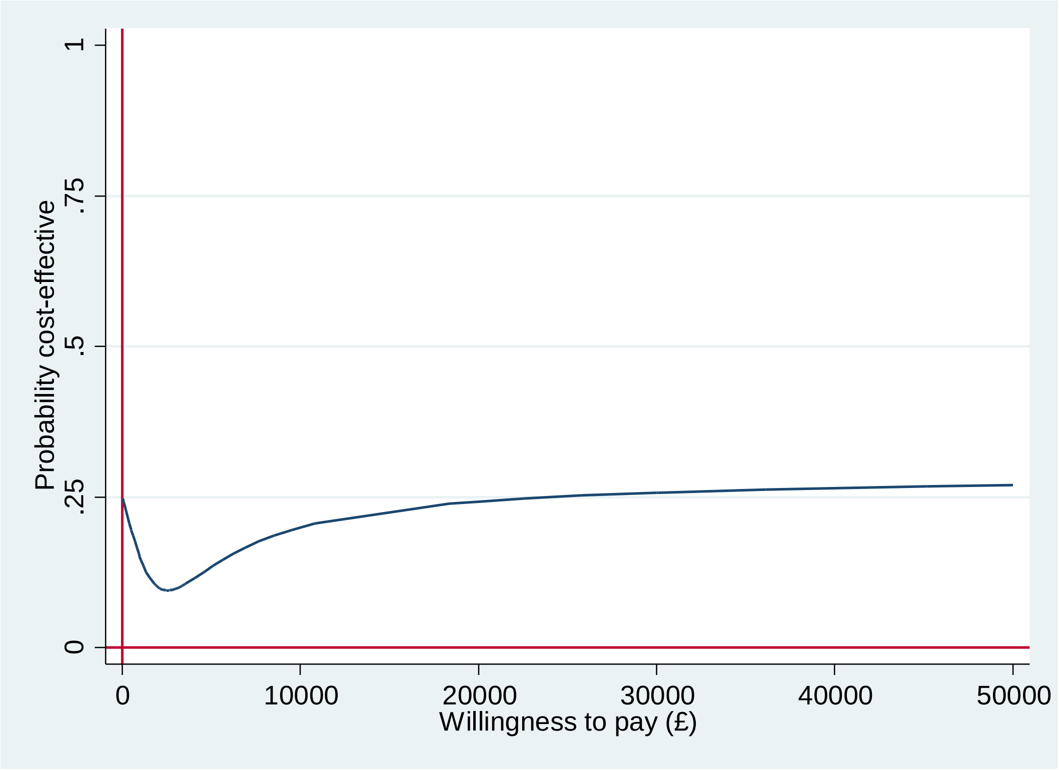

Results of sensitivity analysis 2: One aflibercept injection

Supplementary Table A.7: Care pathway costs – sensitivity analysis 2

Lesion status assessment		Obs[1] (%)	Pathway cost[2] (£) Mean (SD)
**Experts (true)**	**Optometrists' decision**		
Reactivated	Reactivated	795 (39.43)	£1009.24 (45.50)
Reactivated	Suspicious	142 (7.04)	£103.61 (18.51)
Reactivated	Quiescent	57 (2.83)	£51.29 (9.08)
Suspicious	Reactivated	10 (0.50)	£118.12 (16.39)
Suspicious	Suspicious	11 (0.55)	£57.04 (9.10)
Suspicious	Quiescent	14 (0.69)	£52.96 (9.37)
Quiescent	Reactivated	105 (5.21)	£117.14 (32.61)
Quiescent	Suspicious	234 (11.61)	£78.31 (11.53)
Quiescent	Quiescent	648 (32.14)	£51.98 (8.23)
**Experts (true)**	**Ophthalmologists' decision**		
Reactivated	Reactivated	736 (36.51)	£956.50 (46.41)
Reactivated	Suspicious	196 (9.72)	£153.18 (92.25)
Reactivated	Quiescent	62 (3.08)	£77.01 (45.49)
Suspicious	Reactivated	1 (0.05)	£2452.74 (N/A)
Suspicious	Suspicious	17 (0.84)	£68.84 (31.004)
Suspicious	Quiescent	17 (0.84)	£60.57 (17.16)
Quiescent	Reactivated	35 (1.73)	£2493.45 (65.87)

Quiescent	Suspicious	146 (7.24)	£150.34 (95.19)
Quiescent	Quiescent	806 (39.98)	£75.28 (44.72)

[1]The number of observations (i.e. vignettes) is 4032, namely 2016 referring to Optometrists and 2016 referring to Ophthalmologists.
[2]Pathway cost includes the cost of a monitoring consultation and downstream costs (e.g. injections and follow-up visits)

Supplementary Table A.8: Cost-effectiveness – Cost-effectiveness of a monitoring review performed by optometrists versus a monitoring review performed by ophthalmologists – sensitivity analysis 2

**Costs and effects**	**Optometrists Mean (SD) (n=2016)**	**Ophthalmologists Mean (SD) (n=2016)**
Cost of a monitoring review (pathway cost)	£439.90 (460.90)	£425.61 (422.93)
Percentage of correct assessments	84.4% (36.3%)	85.4% (35.3%)
Incremental cost (95% CI)	£14.29 (-£19.91, £48.49)	
Incremental benefit, percentage of correct assessments (95% CI)	-1.0% (-4.5%, 2.5%)	
Incremental cost per correct assessment[1]	Optometrist-led care is dominated	

[1]The 95% CI around the ICER could not be defined

Supplementary Figure A.4 – Optometrists vs opththalmogists cost-effectiveness plane for sensitivity analysis 2, showing the outer 95% confidence ellipse in blue and the 84% confidence ellipse (the widest confidence interval around the ICER that is defined) in brown. The green line shows the tangent to the 84% confidence ellipse.

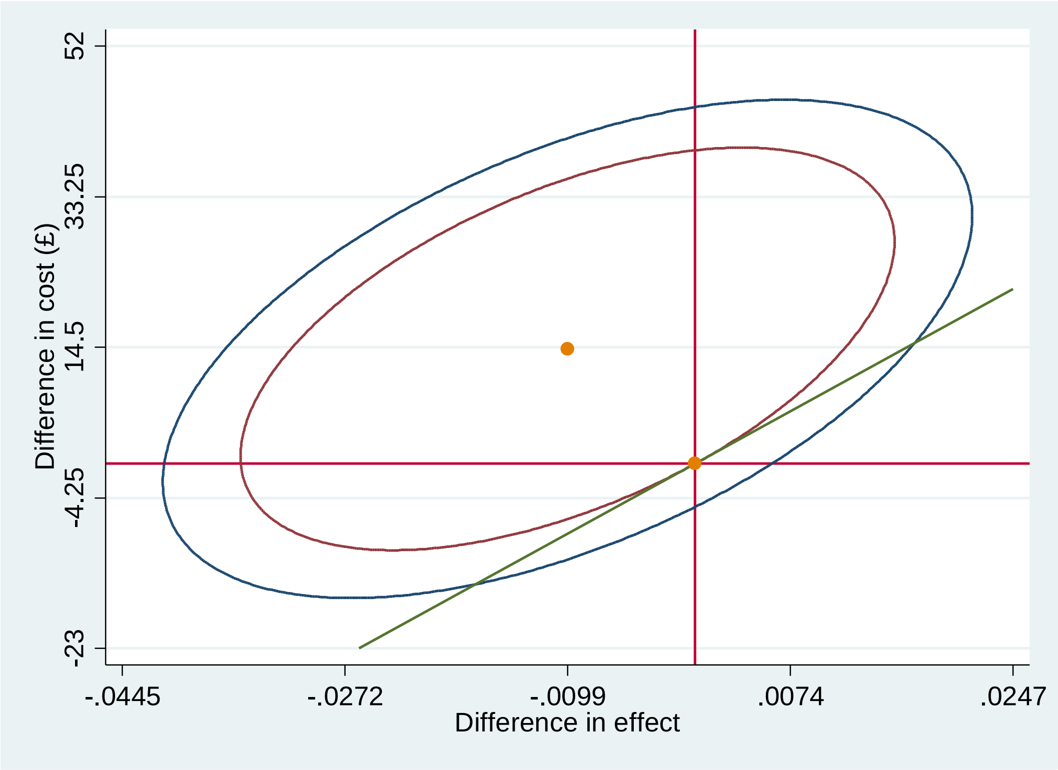

Supplementary FigureA.5: Cost-effectiveness acceptability curve showing the probability that the intervention is cost-effective at different willingness-to-pay thresholds – Optometrists versus Ophthalmologists - sensitivity analysis 2

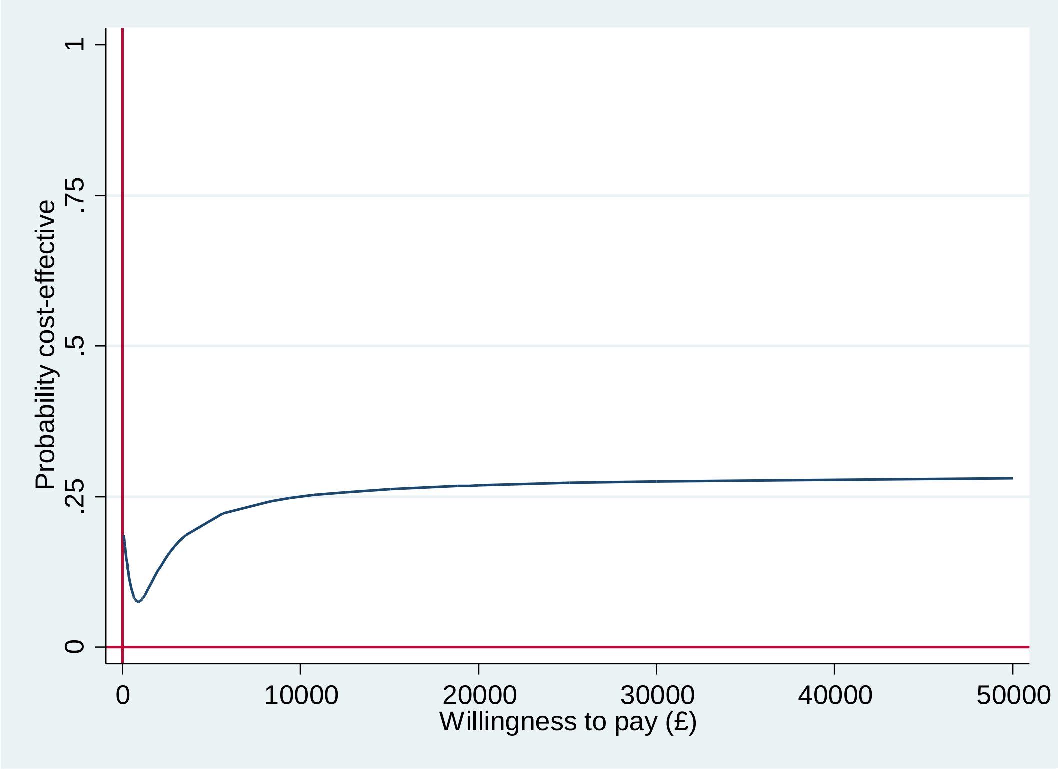

Results of sensitivity analysis 3: One bevacizumab injection

Supplementary Table A.9: Care pathways costs – sensitivity analysis 3

Lesion status assessment		Obs[1] (%)	Pathway cost[2] (£) Mean (SD)
**Experts (true)**	**Optometrists' decision**		
Reactivated	Reactivated	795 (39.43)	£242.23 (45.50)
Reactivated	Suspicious	142 (7.04)	£103.61 (18.51)
Reactivated	Quiescent	57 (2.83)	£51.29 (9.08)
Suspicious	Reactivated	10 (0.50)	£118.12 (16.39)
Suspicious	Suspicious	11 (0.55)	£57.04 (9.10)
Suspicious	Quiescent	14 (0.69)	£52.96 (9.37)
Quiescent	Reactivated	105 (5.21)	£117.14 (32.61)
Quiescent	Suspicious	234 (11.61)	£78.31 (11.53)
Quiescent	Quiescent	648 (32.14)	£51.98 (8.23)
**Experts (true)**	**Ophthalmologists' decision**		
Reactivated	Reactivated	736 (36.51)	£189.50 (46.41)
Reactivated	Suspicious	196 (9.72)	£153.18 (92.25)
Reactivated	Quiescent	62 (3.08)	£77.01 (45.49)
Suspicious	Reactivated	1 (0.05)	£184.21 (N/A)
Suspicious	Suspicious	17 (0.84)	£68.84 (31.004)
Suspicious	Quiescent	17 (0.84)	£60.57 (17.16)
Quiescent	Reactivated	35 (1.73)	£189.12 (38.002)
Quiescent	Suspicious	146 (7.24)	£150.34 (95.19)
Quiescent	Quiescent	806 (39.98)	£75.28 (44.72)

[1]The number of observations (i.e. vignettes) is 4032, namely 2016 referring to Optometrists and 2016 referring to Ophthalmologists.
[2]Pathway cost includes the cost of a monitoring consultation and downstream costs (e.g. injections and follow-up visits)

Supplementary Table A.10: Cost-effectiveness – Cost-effectiveness of a monitoring review performed by optometrists versus a monitoring review performed by ophthalmologists – sensitivity analysis 3

**Costs and effects**	**Optometrists Mean (SD) (n=2016)**	**Ophthalmologists Mean (SD) (n=2016)**
Cost of a monitoring review (pathway cost)	£137.43 (91.78)	£131.89 (77.12)
Percentage of correct assessments	84.4% (36.3%)	85.4% (35.3%)
Incremental cost (95% CI)	£5.54 (-£0.834, £11.916)	
Incremental benefit, percentage of correct assessments (95% CI)	-1.0% (-4.5%, 2.5%)	
Incremental cost per correct assessment	Optometrist-led care is dominated	

Supplementary Figure A.6 – Optometrists vs opththalmogists cost-effectiveness plane for sensitivity analysis 3, showing the outer 95% confidence ellipse in black and the 50% confidence ellipse in purple. The red and blue lines show the tangent to the 95% confidence ellipse.

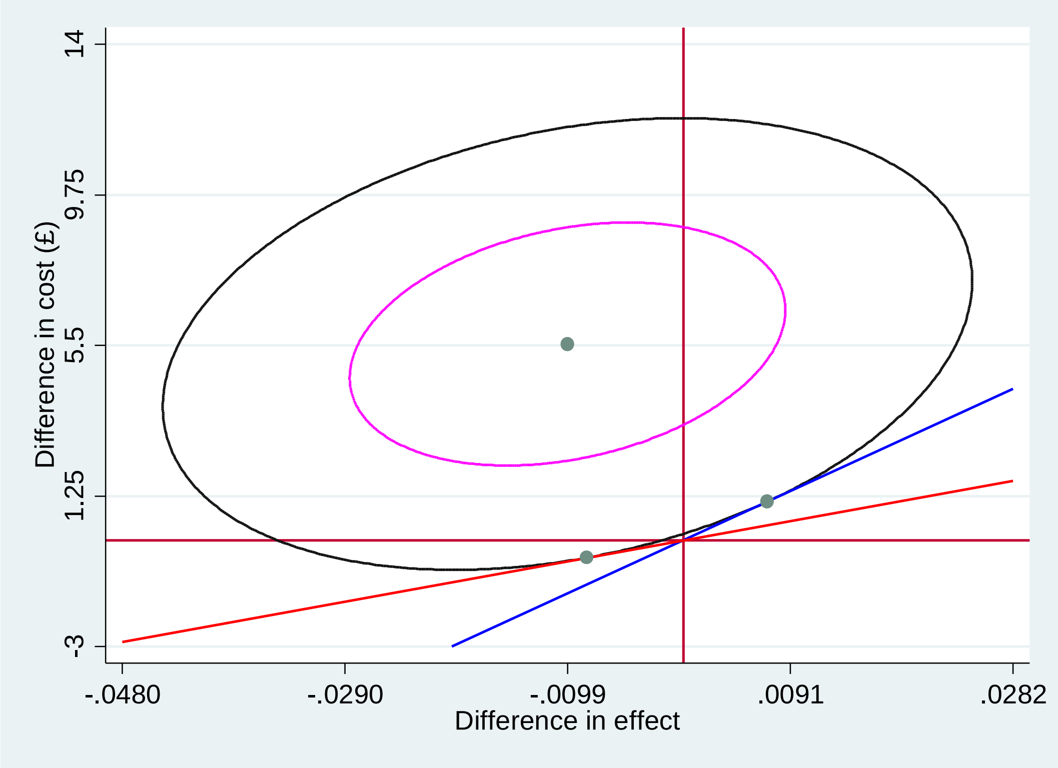

Supplementary Figure A.7: Cost-effectiveness acceptability curve showing the probability that the intervention is cost-effective at different willingness-to-pay thresholds – Optometrists versus Ophthalmologists - sensitivity analysis 3

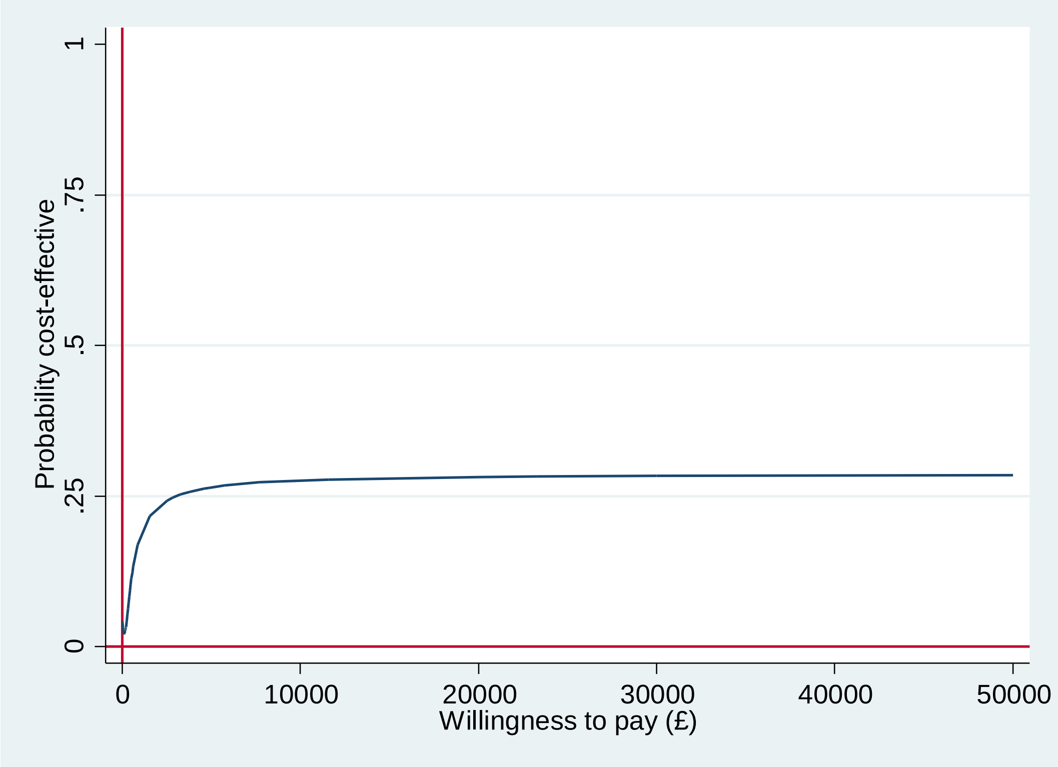

Sensitivity analysis 4

This sensitivity analysis considers only the cost of a monitoring review rather than considering the cost of the whole pathway (**Supplementary Table A.11**). As **Supplementary Table A.12** shows, this analysis finds optometrist-led care to be less effective and significantly less costly than ophthalmologist-led care. Ophthalmologist-led care costs £2389 per additional correct treatment decision compared with optometrist-led care. Even though the maximum amount the NHS is willing to pay for a correct retreatment decision is unknown, it is unlikely to be so high as £2389, which would suggest that optometrist-led care is good value for money if you focus exclusively on the cost of the initial monitoring consultation and ignore downstream costs. As shows, at ceiling ration of £600 or lower, we can be 95% confident that optometrists are a cost-effective option compared with ophthalmologist in this fourth sensitivity analysis. This result emphasises the importance of building a simple decision model to explore the consequences after the initial monitoring review, rather than using only the information from the initial review. Furthermore, it also indicates that the conclusions of the analysis may be sensitive to the assumptions within the decision tree.

Supplementary Table A.11: Care pathway costs – sensitivity analysis 4

Lesion status assessment		Obs[1] (%)	Pathway cost[2] (£) Mean (SD)
**Experts (true)**	**Optometrists' decision**		
Reactivated	Reactivated	795 (39.43)	£51.79 (8.49)
Reactivated	Suspicious	142 (7.04)	£51.81 (9.26)
Reactivated	Quiescent	57 (2.83)	£51.29 (9.08)
Suspicious	Reactivated	10 (0.50)	£50.64 (7.12)
Suspicious	Suspicious	11 (0.55)	£57.04 (9.10)
Suspicious	Quiescent	14 (0.69)	£52.96 (9.37)
Quiescent	Reactivated	105 (5.21)	£51.49 (8.01)
Quiescent	Suspicious	234 (11.61)	£52.21 (7.69)
Quiescent	Quiescent	648 (32.14)	£51.98 (8.23)
**Experts (true)**	**Ophthalmologists' decision**		
Reactivated	Reactivated	736 (36.51)	£76.09 (43.66)
Reactivated	Suspicious	196 (9.72)	£76.59 (46.13)
Reactivated	Quiescent	62 (3.08)	£77.01 (45.49)
Suspicious	Reactivated	1 (0.05)	£89.70 (N/A)
Suspicious	Suspicious	17 (0.84)	£68.84 (31.004)
Suspicious	Quiescent	17 (0.84)	£60.57 (17.16)
Quiescent	Reactivated	35 (1.73)	£76.71 (38.22)
Quiescent	Suspicious	146 (7.24)	£75.17 (47.59)
Quiescent	Quiescent	806 (39.98)	£75.28 (44.72)

[1]The number of observations (i.e. vignettes) is 4032, namely 2016 referring to Optometrists and 2016 referring to Ophthalmologists.
[2]Pathway cost includes the cost of a monitoring consultation and downstream costs (e.g. injections and follow-up visits)

Supplementary Table A.12: Cost-effectiveness – Cost-effectiveness of a monitoring review performed by optometrists versus a monitoring review performed by ophthalmologists – sensitivity analysis 4

Costs and effects	Optometrists Mean (SD) (n=2016)	Ophthalmologists Mean (SD) (n=2016)
Cost of a monitoring review	£51.90 (8.36)	£75.60 (44.31)
Percentage of correct assessments	84.4% (36.3%)	85.4% (35.3%)
Incremental cost (95% CI)	-£23.70 (-£26.09,-£21.31)	
Incremental benefit, percentage of correct assessments (95% CI)	-1.0% (-4.5%, 2.5%)	
Incremental cost per correct assessment[1]	£2389.07	

Supplementary Figure A.8 – Optometrists vs opththalmogists cost-effectiveness plane for sensitivity analysis 4, showing the outer 95% confidence ellipse in black and the 50% confidence ellipse in purple. The blue and red lines are the lower and upper confidence limits, respectively, of the 95% confidence ellipse.

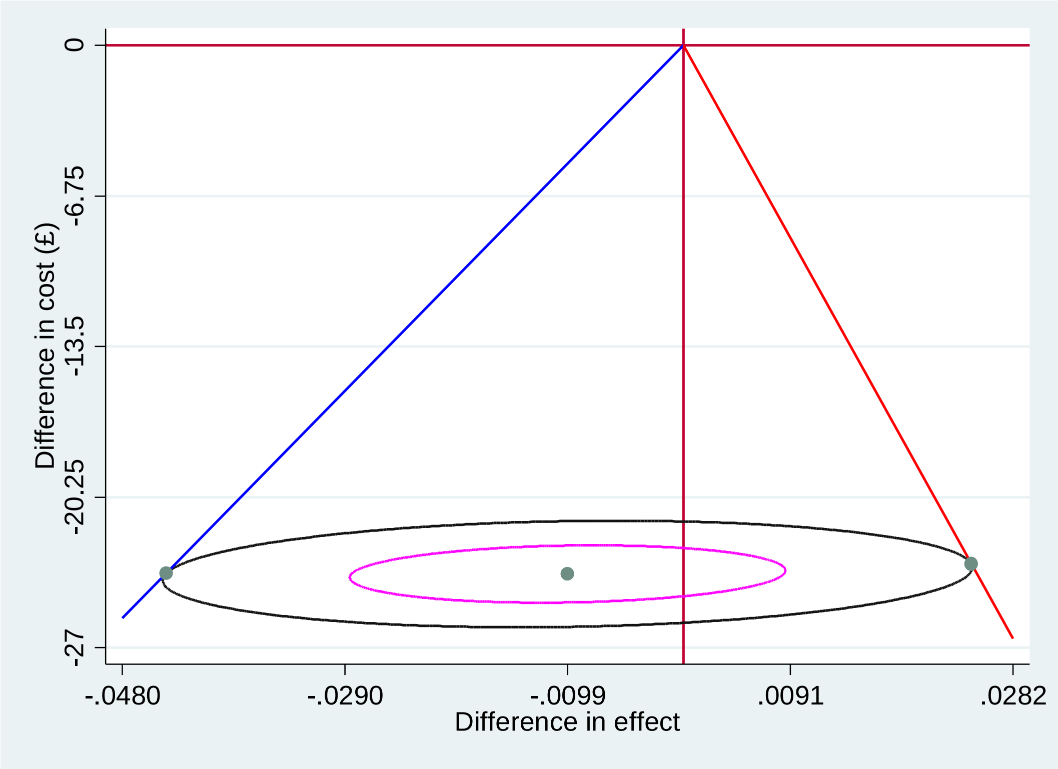

Supplementary FigureA.9: Cost-effectiveness acceptability curve showing the probability that the intervention is cost-effective at different willingness-to-pay thresholds – Optometrists versus Ophthalmologists - sensitivity analysis 4

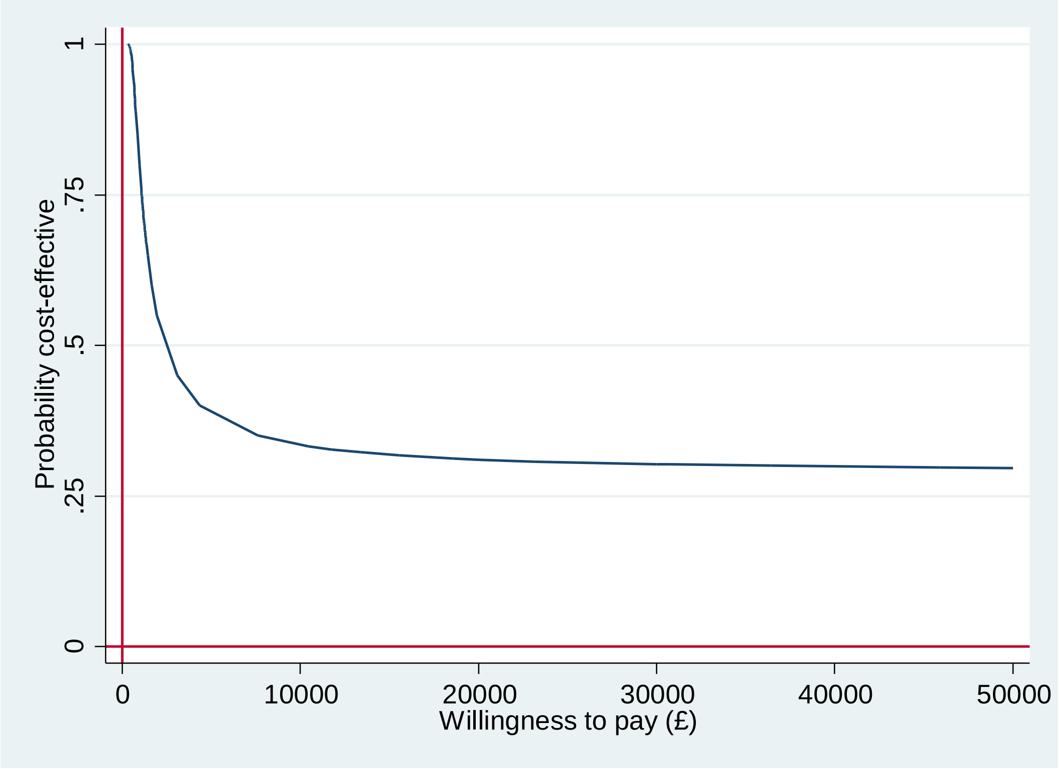

Sensitivity Analysis 5
This sensitivity analysis consisted in modifying the base-case scenario to account for a different 'care pathways model' and associated costs. In particular, whenever the optometrist's judgment was 'reactivation', we assumed that the OCT images taken by the optometrist would be further analysed by an ophthalmologist with the aim to confirm or not the optometrist's assessment. In the base-case analysis the assumption was instead that the patient would have undergone another full monitoring visit at the eye hospital prior to treatment. Based on the expert opinion of the clinicians in the ECHoES trial team, the average duration of this further ophthalmologic check was set to 16.5 minutes. The associated cost was estimated based on the hourly rate of ophthalmologist's time as reported in the IVAN trial [3], and added to the cost and cost-effectiveness analyses.

Supplementary Table A.13 below shows that when optometrists correctly judge the lesion as 'reactivated', the average pathway cost for optometrists is lower than in the base case analysis (see Table 1 in the main article), because in this new care model we have assumed that patients do not need to be referred for another complete monitoring review at the hospital eye clinic before actually receiving treatment, but instead it will suffice for the ophthalmologist to review the OCT images taken by the optometrist to assess the accuracy of the optometrist's judgement. Similarly, when vignettes are truly suspicious/quiescent and optometrists assess the lesion as 'reactivated', the average pathway cost for optometrists is lower than the corresponding value in the base case analysis.

Supplementary Table A.13 : Care pathway costs – sensitivity analysis 5

Lesion status assessment		Obs[1] (%)	Pathway cost[2] (£) Mean (SD)
**Experts (true)**	**Optometrists' decision**		
Reactivated	Reactivated	795 (39.43)	£885.13 (19.80)
Reactivated	Suspicious	142 (7.04)	£103.61 (18.51)
Reactivated	Quiescent	57 (2.83)	£51.29 (9.08)
Suspicious	Reactivated	10 (0.50)	£77.27 (7.12)
Suspicious	Suspicious	11 (0.55)	£57.04 (9.10)
Suspicious	Quiescent	14 (0.69)	£52.96 (9.37)
Quiescent	Reactivated	105 (5.21)	£78.12 (8.01)
Quiescent	Suspicious	234 (11.61)	£78.31 (11.53)
Quiescent	Quiescent	648 (32.14)	£51.98 (8.23)
**Experts (true)**	**Ophthalmologists' decision**		
Reactivated	Reactivated	736 (36.51)	£882.67 (46.41)
Reactivated	Suspicious	196 (9.72)	£153.18 (92.25)
Reactivated	Quiescent	62 (3.08)	£77.01 (45.49)
Suspicious	Reactivated	1 (0.05)	£877.38 (n/a)
Suspicious	Suspicious	17 (0.84)	£68.84 (31.004)
Suspicious	Quiescent	17 (0.84)	£60.57 (17.16)
Quiescent	Reactivated	35 (1.73)	£882.29 (38.002)
Quiescent	Suspicious	146 (7.24)	£150.34 (95.19)
Quiescent	Quiescent	806 (39.98)	£75.28 (44.72)

[1]The number of observations (i.e. vignettes) is 4032, namely 2016 referring to Optometrists and 2016 referring to Ophthalmologists.
[2]Pathway cost includes the cost of a monitoring consultation and downstream costs (e.g. injections and follow-up visits)

When the new care pathway model is used, in conjunction with health outcomes, to investigate the cost-effectiveness of a monitoring review performed by optometrists versus a monitoring review performed by ophthalmologists, the results are overall in line with what already found for the base case scenario (see Table 1 in the main article). Once again, in fact, we find a situation in which no statistically significant difference is observed either in costs or effects.

Supplementary Table A.14 below shows that the differences in costs and in effects between the two professional groups are still small and not statistically significant, but the optometrist-led reviews now decrease the total costs by £8.61 per review (in the base case-scenario there was an increase of £13): a decrease of 2% of the total cost of the care pathway (vs. a 3% increase in the base case scenario). As in the base case scenario, optometrist-led reviews result in only one more incorrect decision per 101 monitoring reviews conducted. As result, in this scenario optometrist-led care would save £870 every additional incorrect retreatment decision. In other words, ophthalmologists-led monitoring reviews costs an additional £870 per correct re-treatment decision compared with optometrist-led care. Referring patients to receive monitoring at community optometry practices is therefore cost-effective if the NHS is willing to accept one additional incorrect retreatment decision in order to save £870.

In this new sensitivity analysis, there is still substantial uncertainty around the finding, as in the base case scenario. The CEAC in **Supplementary Figure A.5** below finds a much higher probability that optometrist-led monitoring reviews are cost-effective. If the NHS is willing to pay £200 per correct retreatment decision, the probability that it is cost-effective to conduct monitoring by community optometrists is 68.5% (the corresponding probability in the base-case scenario was 14%), which

decreases to a probability of 58% if the NHS were willing to pay £600 per correct decision (the corresponding probability in the base-case scenario was 8%). As the willingness to pay threshold increases, the probability that optometrist-led reviews are cost-effectiveness steeply decreases, then stabilises at around 30% for willingness to pay values higher than £5000.

Since the difference in cost between monitoring reviews conducted by optometrists and ophthalmologists was not statistically significant, we can be no more than 70% confident that optometrist-led and ophthalmologist-led care is cost-effective. However, this analysis nonetheless suggests that optometrist-led monitoring reviews would be cost-effective compared with ophthalmologist-led reviews if the HES relied upon re-review of the OCT images taken by optometrists, rather than conducting an additional monitoring review for all patients referred by optometrists.

Supplementary Table A.14: Cost-effectiveness of a monitoring review performed by optometrists versus a monitoring review performed by ophthalmologists – sensitivity analysis 5

	Optometrists Mean (SD) (n=2016)	Ophthalmologists Mean (SD) (n=2016)
Cost of a monitoring review (pathway cost)	£388.73 (401.18)	£397.33 (387.46)
Percentage of correct assessments	84.4% (36.3%)	85.4% (35.3%)
Incremental cost (95% CI)	-£8.61 (-£38.74, £21.52)	
Incremental benefit, percentage of correct assessments (95% CI)	-1.0% (4.5%, 2.5%)	

[1]The 95% CI around the ICER could not be defined

Supplementary Figure A.10 – Optometrists vs opththalmogists cost-effectiveness plane for sensitivity analysis 5 (inner ellipse: 48%; outer ellipse: 95%) showing the outer 95% confidence ellipse in blue and the 48% confidence ellipse (the widest confidence interval around the ICER that is defined) in brown. The green line shows the tangent to the 48% confidence ellipse.

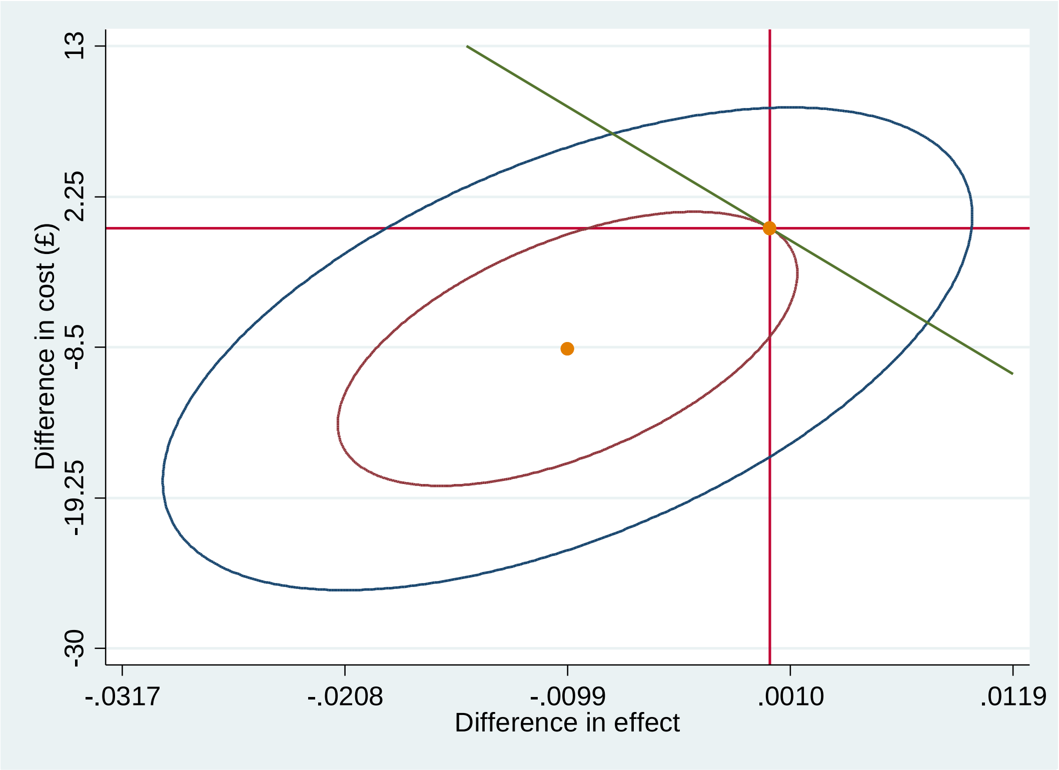

Supplementary Figure A.11: Cost-effectiveness acceptability curve showing the probability that the intervention is cost-effective at different willingness-to-pay thresholds – Optometrists versus Ophthalmologists - sensitivity analysis 5

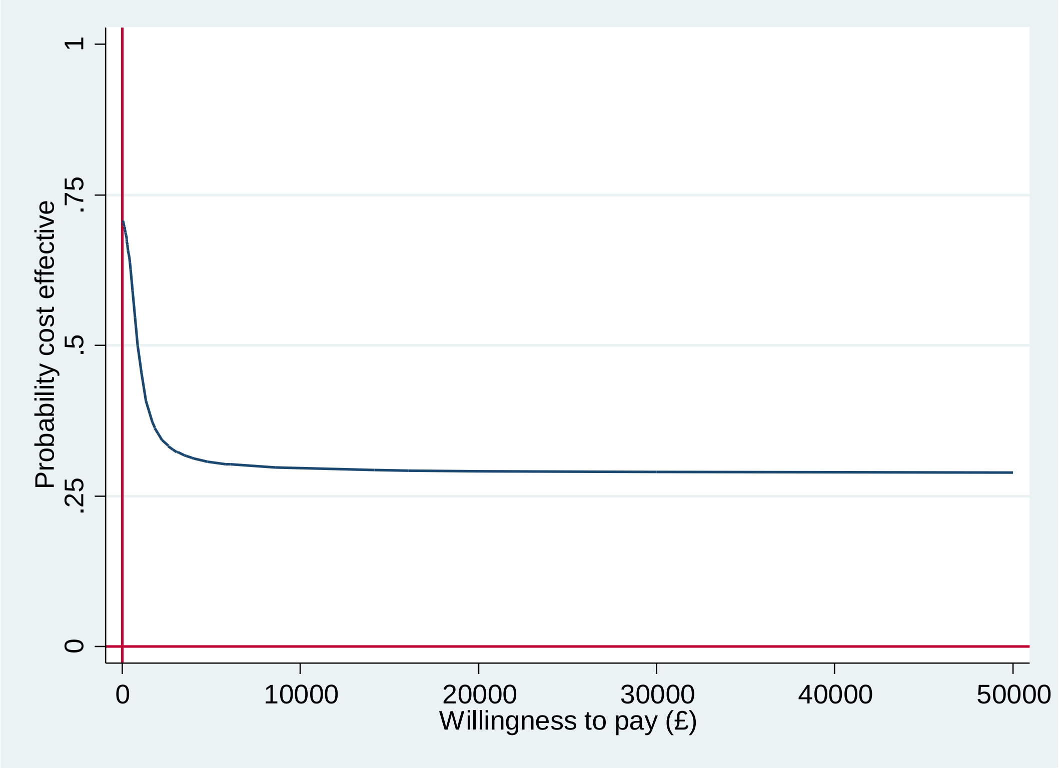

REFERENCES

1. Joint Formulary Commitee. British National Formulary (online) London: BMJ Group and Pharmaceutical Press, 2014.
2. Curtis L. Unit Costs of Health and Social Care 2013. Canterbury: PSSRU, University of Kent, 2013.
3. Dakin HA, Wordsworth S, Rogers CA, et al. Cost-effectiveness of ranibizumab and bevacizumab for age-related macular degeneration: 2-year findings from the IVAN randomised trial. *BMJ open* 2014;**4**(7):e005094 doi: 10.1136/bmjopen-2014-005094

Supplementary material – Resource use and cost questionnaire

The **E**ffectiveness of **C**ommunity vs. **Ho**spital **E**ye **S**ervice follow-up for patients with neovascular age-related macular degeneration with quiescent disease: a virtual trial.

RESOURCE USE AND COST QUESTIONNAIRE FOR OPTOMETRISTS

The **E**ffectiveness of **C**ommunity vs. **Ho**spital **E**ye **S**ervice follow-up for patients with neovascular age-related macular degeneration with quiescent disease: a virtual trial.

ECHoES
Effectiveness of Community vs. Hospital Eye Service

RESOURCE USE AND COST QUESTIONNAIRE FOR OPTOMETRISTS

Date..............................(*please fill in*)

Thank you for taking time to complete this questionnaire. Your answers will be kept confidential and used only for the ECHoES study.

One component of the ECHoES study is to provide the NHS with information on how cost-effective it is for optometrists to perform retreatment assessments for patients with quiescent neovascular age-related macular degeneration (nAMD).

In order to do this we would like you to answer, to the best of your knowledge, the following questions about any resources and costs that would be associated with providing this new service in optometric practices.

Notes on questionnaire completion:

- If you do not know the answer to any given question, please give your best guess wherever possible and otherwise leave it blank and complete the rest of the questionnaire.
- Please write "0" in response to any questions to which the answer is zero.
- All costs should **include VAT** whenever applicable.
- Questions will be reported in Italics, while explanatory text to set the background to the question will be reported in normal font.

If you have any questions regarding this questionnaire please contact:
Dr Mara Violato (mara.violato@dph.ox.ac.uk; Tel 01865 289265) or
Dr Sarah Wordsworth (sarah.wordsworth@dph.ox.ac.uk; Tel 01865 289268)

****
Set up/ Capital and operative costs
****

Imagine that the community optometric practice where you work is going to be involved in shared care-management for monitoring the need for retreatment of patients with nAMD whose disease has been rendered quiescent by anti-VEGF drugs, such as Avastin or Lucentis. We would like to know which facilities the practice already has and which facilities need to be set up in order to be able to offer this new monitoring service.

When answering, please keep in mind that the appointment for monitoring will typically consist of the components and skills summarised in Table 1:

Table 1

**Component**	**Description**	**Skills required**
History	Discussion of patient-reported vision status in each eye and comparison to status at previous visit.	Communication skills
Clinical examination<br>Slit lamp biomicroscopy:<br>Anterior segment and macula	Clinical exam to ensure absence of VEGF-related adverse events and/or incidental other disease.	Slit lamp and ophthalmoscopy skills
Visual acuity assessment	Visual acuity recorded as letters read on an ETDRS chart at 4 metres (with/without mirrors) using previously recorded refraction.<br><br>The results will then be recorded in the patient medical record.	Test and interpret visual acuity
Administration of 1% tropicamide drops	Pupil dilation. Drops will need to be administered 20 minutes before colour fundus photography and spectral domain coherence tomography.	Instillation of eye drops
Colour fundus photography (or equivalent colour fundus image)	One good quality photograph centred on the centre of the macula of each eye.	Taking and interpreting retinal images
Spectral domain optical coherence tomography (OCT)	Cube scan of the posterior pole for each eye. Images will be acquired using a standardised protocol, which is pre-set on the OCT machine.	Taking and interpreting OCT images
Final assessment	A retreatment decision will be made on the basis of the visual acuity data and interpretation of images obtained.<br><br>The decision and rationale will need to be entered in the patient record.	Ability to assess the need for retreatment and arrange necessary follow-up

Q1. *Given the above description, how long do you think each monitoring review will take?* Please include in your estimate the 20 minutes that the patient will have to wait for the dilating drops to work.

.....minutes

As optometric practices are very heterogeneous, we would first like to have a sense of such diversity in order to plan a shared-care programme that accounts for the needs of all practices. We would therefore like you to describe your practice by replying to the following questions.
N.B. If you own/work in more than one practice, please reply to the whole questionnaire referring only to the practice where you spend most of your time.

Q2. *What is the approximate size of the practice you work in?*
(Please answer ONLY ONE of the alternative options)

- ☐ *... square metres*
- ☐ *... square feet*
- ☐ *I am unsure*

Q3. *How many rooms are there in your practice?*

... rooms

Q4. *What facilities are available in the practice you work in that could be used to assess visual function and/or perform optometric monitoring on patients with nAMD?*
N.B. If the practice does not have a specific piece of equipment please type "0" in the corresponding line. **Go to page 2 to see again Table 1 describing a typical monitoring review.**

**Equipment**	**Quantity currently in the practice**
ETDRS visual acuity charts - 4 metres viewing distance required (with/without mirrors)	
Projector that includes ETDRS chart	
Retro-illuminated light box	
Trial frame	
Lens set	
Light meter to measure luminance. e.g. SPER Scientific	
Slit-lamp	
Colour fundus camera	
OCT acquisition system	
OCT acquisition system with fundus photography included as a component	
Computer	
Do you have a computer network (allowing images to be viewed at more than one workstation)?	**YES/NO**
Colour fundus camera software	
OCT software	
Printer	
Other (please specify)	

PLEASE COMPLETE ALSO QUESTIONS Q4.a, Q4.b and Q4.c AS APPROPRIATE IF YOUR PRACTICE HAS ANY OF THE FOLLOWING:

Colour fundus camera

OCT acquisition system

OCT acquisition system with fundus photography included as a component

Q4.a

"Colour fundus camera"

Approximately when was the latest colour fundus camera acquired?

…. years and …. months ago

What model is the colour fundus camera that you most recently acquired?

……

…….

How many patients currently use the colour fundus camera per week?…………………..

How much did the colour fundus camera cost?

(Please choose and complete only one of the following options)

Costs should include VAT

- ☐ *£……………… one off purchase AND £………….. per year for service/maintenance/call out fee*
- ☐ *£…………….. per month hire AND £………………. per year service/maintenance/call out fee*
- ☐ *£…………….. per month hire INCLUDING service/maintenance/call out fee*
- ☐ *I am unsure*

Q4.b

"OCT acquisition system"

Approximately when was the latest OCT acquisition system acquired?

…. years and …. months ago

What model is the OCT that you most recently acquired?

…….

…….

How many patients currently use the OCT acquisition system per week?……………………….

How much did the OCT acquisition system cost?

(Please choose and complete only one of the following options)

Costs should include VAT

- ☐ *£……………… one off purchase AND £….. per month service/maintenance/call out fee*
- ☐ *£……………… per month hire AND £….. per month service/maintenance/call out fee*
- ☐ *£……………… per month hire INCLUDING service/maintenance/call out fee*
- ☐ *I am unsure*

Q4.c

"OCT acquisition system with added colour fundus photography feature"

Approximately when was the most recent OCT acquisition system with added colour fundus photography feature acquired?

…. years and …. months ago

What model is the OCT acquisition system with added colour fundus photography that you most recently acquired?
……….
……….

How many patients currently use the OCT acquisition system with added colour fundus photography per week?……………………………………

How much did the OCT acquisition system with added colour fundus photography feature cost to buy and how much is maintenance?
(Please choose and complete only one of the following options)

Costs should include VAT

- ☐ *£……………… one off purchase AND £….. per month service/maintenance/call out fee*
- ☐ *£……………… per month hire AND £….. per month service/maintenance/call out fee*
- ☐ *£……………… per month hire INCLUDING service/maintenance/call out fee*
- ☐ *I am unsure*

Q5. *We would like now to know about the staff (including yourself) in your practice, their role, working hours, average salary and how long they would potentially spend on each monitoring review to perform retreatment assessments for patients with quiescent nAMD. Please complete the table below to the best of your knowledge.* If a staff type indicated in the table is not employed at your practice, then leave the corresponding row blank. If there is more than one staff for each type, please fill in a row for each (e.g. Optometrist 1, Optometrist 2, etc…). **Go to page 2 to see again Table 1 describing a typical monitoring review. If you need additional space, please give further details on a separate piece of paper and label it as "Q5".**

For replies in columns 2 and 5, please use one of the categories indicated below the table.

Table Q5

**Staff type**	**Role that they would have during the consultation (please choose one or more from the list below[a])**	**Time that they would spend on patient-related activities per monitoring review (minutes)** Please refer back to the total consultation length that you reported in your reply Q1	**Hours worked per week**	**Annual salary (gross) band[b]**
Optometrist 1: yourself				
Pre-registration optometrist 1				

[a] Please use one or more of the following categories in column 2:
1. History
2. Clinical examination
3. Visual acuity assessment
4. Administration of eye drops
5. Colour fundus photography
6. OCT
7. Assessment of need for retreatment and next follow-up
8. Updating patient records
9. Booking appointments
10. None of the above
11. Other (please specify)

[b] Please give the salary in one of the following bands: Less than £20,000; £20,000 to £29,999; £30,000 to £39,999; £40,000 to £49,999; £50,000 to £59,999; £60,000 to £69,999; £70,000 to £79,999; £80,000 per year or more.

Staff type	Role that they would have during the consultation **<u>(please choose one or more from the list below[a])</u>**	Time that they would spend on patient-related activities per monitoring review (minutes) Please refer back to the total consultation length that you reported in your reply Q1	Hours worked per week	Annual salary (gross) band[b]
Optical assistant 1				
Clerical/retailer staff 1				
Practice manager				

Staff type	Role that they would have during the consultation <u>(please choose one or more from the list below[a])</u>	Time that they would spend on patient-related activities per monitoring review (minutes) Please refer back to the total consultation length that you reported in your reply Q1	Hours worked per week	Annual salary (gross) band[b]
Other administrative staff 1				
Other (specify)				

**Staff type**	**Role that they would have during the consultation <u>(please choose one or more from the list below[a])</u>**	**Time that they would spend on patient-related activities per monitoring review (minutes)** **Please** refer back to the total consultation length that you reported in your reply Q1	**Hours worked per week**	**Annual salary (gross) band[b]**

[a] Please use one or more of the following categories in column 2:

1. History
2. Clinical examination
3. Visual acuity assessment
4. Administration of eye drops
5. Colour fundus photography
6. OCT
7. Assessment of need for retreatment and next follow-up
8. Updating patient records
9. Booking appointments
10. None of the above
11. Other (please specify)

[a] Please give the salary in one of the following bands: Less than £20,000; £20,000 to £29,999; £30,000 to £39,999; £40,000 to £49,999; £50,000 to £59,999; £60,000 to £69,999; £70,000 to £79,999; £80,000 per year or more.

We anticipate that for any given monitoring visit, each individual task will be conducted by one individual optometrist. If that is not the case could you, please give details of which tasks would be done jointly by two or more people working on the same patient visit?

………
………
……….
……….

Q6. *Given the CURRENT size and structure of your practice would it be necessary to set up or modify any room(s) to offer the additional monitoring service?*

- ☐ *Yes*
- ☐ *No*

If **YES**

Q6.a *How many rooms would need to be set up/modified?* ……………………

Q6.b *What kind of adjustments would be required to the existing space in your practice?*
(Please tick as many options as appropriate)

- ☐ *It would be necessary to modify an existing room(s) to create the necessary space to offer the new service*
- ☐ *It would be necessary to build a separate room by scaling down the size of another room in the practice*
- ☐ *It would be necessary to rent an additional room adjacent to the practice*
- ☐ *It would be necessary to extend the current size of the practice by building an "extra" room*
- ☐ *Other (please specify)*
  ………
  ………
  ………

Q6.c *With reference to the previous question, what* <u>*kind*</u> *of costs would your practice incur to set/up modify any existing/additional room for providing the monitoring service?*
(Please tick as many options as appropriate)

- ☐ *Refurbishment /conversion – please answer also* ***Q6.d***
- ☐ *Building/construction work– please answer also* ***Q6.e***
- ☐ *Additional rent – please answer also* ***Q6.f***
- ☐ *New OCT acquisition system*
  How many additional OCT systems would be required? …..systems
- ☐ *New colour fundus photography*
  How many additional photography systems would be required? …..systems
- ☐ *New OCT acquisition system with added colour fundus photography*
  How many additional OCT/photography systems would be required? …..systems
- ☐ *Other new specialist equipment (please specify)*
  ………
  ………
  ………
- ☐ *New furniture (please specify)*
  ………
  ………
  ………
- ☐ *Other (please specify)*
  ………
  ………
  ………

If you expect your practice would incur "building/construction work" or "refurbishment /conversion work" or would need to "rent an additional room", how much would that cost (excluding cost for specialist equipment, office equipment and furniture/furnishing)? **Please include VAT in your replies if applicable.**

Q6.d *"Refurbishment /conversion"*
(Please choose and complete only one of the following options)

- ☐ *£................................."one-off" expenditure*
- ☐ *I am unsure*
- ☐ *Other (please specify)*

....
....
....

Q6.e *"Building/construction work"*
(Please choose and complete only one of the following options)

- ☐ *£................................."one-off" expenditure*
- ☐ *I am unsure*
- ☐ *Other (please specify)*

....
....
....

Q6.f *"Additional rent"*
(Please choose and complete only one of the following options)

- ☐ *£.................................per month*
- ☐ *I am unsure*
- ☐ *Other (please specify)*

....
....
....

Q7. *After you have done any modifications to the practice (if needed) and hired any additional staff required (if necessary), how many patients with quiescent nAMD do you think your practice could accommodate per month in addition to your current monthly volume of clients?* Please keep in mind the description of a typical monitoring review as presented at the beginning of the questionnaire **(go to page 2 to see again Table 1)** and the estimated time for each monitoring review in minutes that **you indicated in Q1.**

.....patients per month

How many optometrists would this workload be shared amongst? ....

****

Staff training

****

Q8. *For each skill listed in the table below, please indicate what training you and your colleagues have received or would need to perform retreatment assessments for patients with quiescent nAMD.* **Go to page 2 to see again Table 1** describing a typical monitoring review. Please give as many details about the additional training as possible. If you do not know what training opportunities are available, please leave the last column blank. **If you need additional space, please give further details on a separate piece of paper and label it as "Q8".**

Table Q8

**Skills**	**Do you currently feel confident that you have mastered this skill?**	**Details of the training I or colleagues have already received on this (course type, provider, length, cost, etc…)**<br>**Costs should include VAT**	**Number of staff in your practice (including yourself) who would benefit from further training to perform the monitoring review**	**Details about training required, if known (course type, provider, length, cost, etc…)**<br>**Costs should include VAT**
Slit lamp biomicroscopy	Yes/No			
Conduct test and interpret ETDRS visual acuity assessment	Yes/No			

**Skills**	**Do you currently feel confident that you have mastered this skill?**	**Details of the training I or colleagues have already received on this (course type, provider, length, cost, etc…)**<br>**Costs should <u>include</u> VAT**	**Number of staff in your practice (<u>including yourself</u>) who would benefit from further training to perform the monitoring review**	**Details about training required, <u>if known</u> (course type, provider, length, cost, etc…)**<br>**Costs should <u>include</u> VAT**
Administration of 1% tropicamide drops	Yes/No			
Taking colour fundus photography images	Yes/No			
Interpreting colour fundus photography images	Yes/No			

**Skills**	**Do you currently feel confident that you have mastered this skill?**	**Details of the training I or colleagues have already received on this (course type, provider, length, cost, etc…)**<br>**Costs should <u>include</u> VAT**	**Number of staff in your practice (<u>including yourself</u>) who would benefit from further training to perform the monitoring review**	**Details about training required, <u>if known</u> (course type, provider, length, cost, etc…)**<br>**Costs should <u>include</u> VAT**
Taking OCT images	Yes/No			
Interpreting OCT images	Yes/No			
Making decisions about the need for retreatment and time to next follow-up	Yes/No			

Are you familiar with any electronic patient record systems?

- ☐ *Yes*
- ☐ *No*

If yes, which?

……

……

Would you be willing to use an electronic patient record system in order to offer shared care of quiescent nAMD?

- ☐ *Yes*
- ☐ *No*

****

Final questions

****

Q9. *What would motivate you to join a shared-care scheme? Please choose as many options as applicable.*

(Please tick as many options as appropriate)

- ☐ *To allow the NHS to make a better use of the scarce resources by optometrists sharing the care of quiescent nAMD patients*
- ☐ *Clinical interest*
- ☐ *Career development*
- ☐ *Practice reputation*
- ☐ *Income generation*
- ☐ *Other (please specify)*……………………………………………………………………………………………………

……

……

Q10. *Are there any other costs or resources that your practice would need to incur in order to provide monitoring reviews for nAMD patients that are not mentioned in the questionnaire?*

- ☐ *Yes*
- ☐ *No*

If ***YES***

Please provide details below

……

……

……

……

……

Q11. *If you have any comments on the questionnaire (such as difficulties that you encountered interpreting or finding the information for any specific sections), please record them here.*

……

……

……

……

……
